# Cell therapy centered on IL-1Ra is neuroprotective in experimental stroke

**DOI:** 10.1007/s00401-016-1541-5

**Published:** 2016-02-09

**Authors:** Bettina Hjelm Clausen, Kate Lykke Lambertsen, Frederik Dagnæs-Hansen, Alicia Anne Babcock, Christian Ulrich von Linstow, Michael Meldgaard, Bjarne Winther Kristensen, Tomas Deierborg, Bente Finsen

**Affiliations:** Neurobiology Research, Institute of Molecular Medicine, University of Southern Denmark, J. B. Winsloewsvej 25, 5000 Odense C, Denmark; Experimental Neuroinflammation Laboratory, Department of Experimental Medical Science, Lund University, Lund, Sweden; Department of Biomedicine, University of Aarhus, Aarhus C, 8000 Denmark; Department of Clinical Biochemistry, Gentofte Hospital, University of Copenhagen, Gentofte, 2820 Denmark; Department of Pathology, Institute of Clinical Research, Odense University Hospital, Odense C, 5000 Denmark

**Keywords:** IL-1α/β, Microglia, Macrophages, Inflammation, Focal cerebral ischemia, Human brain

## Abstract

Cell-based therapies are emerging as new promising treatments in stroke. However, their functional mechanism and therapeutic potential during early infarct maturation has so far received little attention. Here, we asked if cell-based delivery of the interleukin-1 receptor antagonist (IL-1Ra), a known neuroprotectant in stroke, can promote neuroprotection, by modulating the detrimental inflammatory response in the tissue at risk. We show by the use of IL-1Ra-overexpressing and IL-1Ra-deficient mice that IL-1Ra is neuroprotective in stroke. Characterization of the cellular and spatiotemporal production of IL-1Ra and IL-1α/β identifies microglia, not infiltrating leukocytes, as the major sources of IL-1Ra after experimental stroke, and shows IL-1Ra and IL-1β to be produced by segregated subsets of microglia with a small proportion of these cells co-expressing IL-1α. Reconstitution of whole body irradiated mice with IL-1Ra-producing bone marrow cells is associated with neuroprotection and recruitment of IL-1Ra-producing leukocytes after stroke. Neuroprotection is also achieved by therapeutic injection of IL-1Ra-producing bone marrow cells 30 min after stroke onset, additionally improving the functional outcome in two different stroke models. The IL-1Ra-producing bone marrow cells increase the number of IL-1Ra-producing microglia, reduce the availability of IL-1β, and modulate mitogen-activated protein kinase (MAPK) signaling in the ischemic cortex. The importance of these results is underlined by demonstration of IL-1Ra-producing cells in the human cortex early after ischemic stroke. Taken together, our results attribute distinct neuroprotective or neurotoxic functions to segregated subsets of microglia and suggest that treatment strategies increasing the production of IL-1Ra by infiltrating leukocytes or microglia may also be neuroprotective if applied early after stroke onset in patients.

**Electronic supplementary material:**

The online version of this article (doi:10.1007/s00401-016-1541-5) contains supplementary material, which is available to authorized users.

## Introduction

Novel cell-based therapies have emerged during the past decade, with several ongoing trials investigating the therapeutic effect of bone marrow (BM) cells [48, 57], including mesenchymal stem cells [37], being a cellular source of IL-1Ra [50], which has anti-inflammatory effects [38] and neuroprotective functions in experimental stroke [43, 46, 52]. An advantage of using cell-based therapies in stroke, next to or in combination with recanalization therapies [22], is that cells, which actively infiltrate the neural parenchyma at the site of injury [56, 59], may be able to modulate the inflammatory response in tissues at risk of being recruited into the expanding infarct. The inflammatory response elicited by stroke is orchestrated as a continuum, driven by both activated microglia and infiltrating leukocytes, both known producers of interleukin-1β (IL-1β) [8, 10, 15, 19]. IL-1Ra is a competitive inhibitor of IL-1(α/β) signaling [6, 20, 39, 60, 61] when mediated through the IL-1 receptor type 1 (IL-1R1) found in low numbers on nearly all cells including cortical neurons [13, 36]. The IL-1R2 acts as a decoy receptor [21], and the IL-1R3, which is also expressed on cortical neurons, lacks an IL-1Ra binding site [54]. Although, it is well documented that IL-1Ra attenuates the pro-inflammatory activities of IL-1(α/β) [6, 20, 39, 60, 61], key information about the cellular production of IL-1Ra and IL-1(α/β), and its orchestration in the tissue at risk in ischemic stroke is still lacking, but pivotal to the development of new therapeutic strategies.

Here, we established the cellular sources of IL-1Ra and IL-1(α/β) in a mouse model of stroke, and examined the neuroprotective and anti-inflammatory effect of therapeutically injected IL-1Ra-producing BM cells. Microglia, not infiltrating leukocytes, were the major sources of IL-1Ra with segregated subsets of microglia producing either IL-1Ra or IL-1β, demonstrating functional heterogeneity of microglia at the site of injury. The use of a radiation BM chimeric approach documented that IL-1Ra-producing leukocytes can be neuroprotective. Therapeutic injection of IL-1Ra-producing BM cells post-stroke amplified microglial production of IL-1Ra and reduced brain levels of IL-1β, collectively leading to smaller infarcts and improved functional outcome. In summary, we found the therapeutic injection of cells with increased IL-1Ra production to be neuroprotective, by mechanisms that involve amplification of the endogenous IL-1Ra production. The relevance of these results for future stroke treatment is emphasized by the demonstration that IL-1Ra is also expressed in the infarcted cortex in autopsies from stroke victims.

## Materials and methods

### Mice

In this study, we used adult male IL-1Ra KO mice that lacked all isoforms of IL-1Ra and heterozygous IL-1Ra-Tg mice carrying a transgene encoding the secreted form of IL-1Ra (sIL-1Ra) under the control of its endogenous promoter (Table S1). Both mouse strains were kindly donated by Dr. Emmet Hirsh (Columbia University, USA) and have previously been described in detail [32, 36]. Wild-type littermate (LM) mice were used as controls. IL-1Ra-KO mice lacked IL-1Ra mRNA in the brain, and IL-1Ra-Tg mice expressed higher levels of IL-Ra in the brain than LM mice [IL-1Ra: 252 ± 170 pg/mg (Tg), and 8 ± 3 pg/mg (LM), (mean ± SD), *n* = 14/group]. Both mouse strains were maintained as colonies at the Laboratory of Biomedicine, University of Southern Denmark, Odense, Denmark. In addition, young adult, male and female homozygous C57BL/6-Tg(UBC-GFP)30Scha/J (Stock No. 004353) (GFP-TG) breeding pairs were purchased from the Jackson Laboratory (Bar Harbour, Maine, USA) and transferred to the Department of Biomedicine, University of Aarhus, where they were kept as a colony. IL-1α/β-KO mice [33] were kindly donated by Dr. Yoichiro Iwakura and used for control tissue. All C57BL/6 mice were purchased from Taconic Ltd. (Denmark). The mice were housed under diurnal lighting conditions and given free access to food and water.

### Human postmortem brain tissue

This study was performed using postmortem brain tissue from 21 stroke cases. Sex, age, infarcted brain area and the age of the infarct are given in Table S2. Four cases are part of a previous study on surfactant protein-D in human ischemic brain tissue [42]. Control tissues included brain, kidney, pancreas, lung, placenta and tonsil (not shown). The study was approved by the Regional Committee on Health Research Ethics from the Region of Southern Denmark (Project-ID S-20080042) and performed at the Department of Pathology, Odense University Hospital, Denmark.

### Experimental stroke

All studies were conducted using young age-matched male mice. Focal cerebral ischemia was induced by either permanent (p) or transient (t) middle cerebral artery occlusion (MCAo). *pMCAo* permanent occlusion of the distal part of the left MCA was performed as detailed in Clausen et al. [10]. *tMCAo* transient occlusion (40 min) of the right MCA was performed using the intraluminal filament placement technique, as detailed in Inacio et al. [35], under 1.5 % isoflurane anesthesia. All studies were conducted as randomized, double-blinded studies, with inclusion of sham-operated mice and unlesioned controls (Ctl). Blood flow and body temperature were monitored using the optical fiber probe (VP10 M200ST, UK) and endoscopic temperature probe (T6a, UK) from Moor Instruments. The study was approved by the Danish Veterinary and Food Administration (J. no. 2011/561-1950). An overview of the different mouse groups used for assessment of the effect of IL-1Ra and IL-1Ra-producing cells on infarct volume is provided in Table S1.

### Irradiation BM chimeric mice

Irradiation BM chimeric mice were generated as detailed in Clausen et al. [10]. All recipient mice were irradiated with a single dose of 9.5 Gy from a ^137^Cs source and BM transplantation into the tail vein was performed within 2 h of whole body irradiation. Recipient mice were allowed to reconstitute for 6 weeks prior to pMCAo. The extent of chimerism was assessed in blood samples taken from C57BL/6 mice reconstituted with BM cells from GFP mice at the time of termination (*n* = 5). Flow cytometry showed that 91.8 ± 0.9 % (mean ± SD) of the circulating leukocytes were CD11b^+^CD45^high^ GFP^+^ leukocytes, indicating a successful reconstitution of the mice.

### BM cells for post-stroke treatment

*Harvesting and transplantation* BM cells were harvested in RPMI medium (Gibco, Life Technologies), and approximately 10^7^ cells were injected into the tail vein of recipient mice 30 min after MCAo [10]. *Cell labelling* A Vybrant^®^ CFDA SE Cell (CFSE) Tracer Kit (V12883, Invitrogen) was used for cell labeling and visualization in situ or by flow cytometry. *Characterization* BM cells harvested from IL-1Ra-Tg and LM mice (*n* = 5/group) were analyzed with regard to cell numbers and cell size using the Scepter 2.0 automated Cell Counter from Millipore. To assure compatibility with the Scepter correlation range (50 × 10^5^–1.5 × 10^6^ cells/ml), 10 µl of BM cell suspension was first counted using a Bürker-Türk counting chamber. Next, 100 µl of a BM cell suspension from IL-1Ra-Tg and LM mice were counted using the Scepter automated cell counter employing 40-μm Scepter sensors (Millipore). Histograms and overlays were computed utilizing the Scepter™ 2.0 Cell Counter and Software Pro system.

### Physiological parameters

*Blood gas analysis* Venous blood samples [4] collected 30 min after BM injection (*n* = 6–8/group). were analyzed for pO_2_/pCO_2_, pH, electrolytes, glucose and lactate. *Temperature* Rectal temperature was measured on all mice before surgery (0 h), before BM injection (30 min) and 1 and 3 h after pMCAo using a temperature probe with a Model Bat 12 unit (Physiotemp, New Jersey, USA). *Body weight* All mice were weighed at 0 h and 1, 3 and 5 days after pMCAo.

### Behavioral test

*Grip strength* The grip strength test was performed as previously detailed [4, 40] with the principal investigator blinded to the treatment. The highest force score of the front paws was recorded pre- and post-surgically, using the grip strength meter from BIOSEP (BIO-GT-3, France). *Hargreaves test* Thermal nociception was tested as detailed [5, 49] using the plantar test device (Ugo Basile, Model 37370, Italy). The investigator was blinded to the treatment. Five measurements were registered for the hind paws pre- and post-surgically, the lowest and highest value was excluded and the mean calculated. The mice were allowed to recover for 15 min between trials.

*Open field test* Locomotor and anxiety-related activities were investigated using the open field test [Box: 45 (W) × 45 (D) × 40 (H) cm] with the investigator blinded to the treatment. This test was conducted as previously detailed [41, 45] over a 10 min period. Movement was recorded automatically, while time to first rear, digging, grooming, jumping, urination and droppings were recorded manually and documented as the number of events.

### Tissue processing

*Fresh (CO*_*2*_*) frozen tissue* Brains were processed into six parallel series of sections (30 μm). Separate series were collected on glass slides and used for infarct size analysis, in situ hybridization (ISH) and immunohistochemistry (IHC) or in Eppendorf tubes and used for qPCR, Western blotting, and ELISA. *qPCR* The tissue was processed as detailed by Clausen et al. [12]. *Flow cytometry* The tissue was processed as detailed by Clausen et al. [10]. *Paraformaldehyde fixed tissue* Brains were fixed and processed into 12 series of sections (16 μm) for histological analysis was performed as detailed by Clausen et al. [10].

### Infarct volume

Infarct size analysis was performed according to Cavalieri´s principle for infarct volume estimation (IFV) as previously described on one series of sections spanning the infarct [4, 27] with blinded assessment of the outcome.

### Quantitative PCR

RNA extraction, cDNA synthesis and qPCR were performed with published primers for IL-1β mRNA and HPRT1 mRNA as described by Clausen et al. [12]. In addition, the following primer sequences were used, all spanning exon–exon junctions with sequence specificity checked using BLAST: IL-1Ra mRNA (sense: AAC CAG CTC ATT GCT GGG TAC TTA; antisense: GCC CAA GAA CAC ACT ATG AAG GTC), IL-1α mRNA (Sense: GTC GGC AAA GAA ATC AAG ATG; antisense GTC TTC GTT TTC ACT GTA ACA G), IL-1R1 mRNA (sense: ACA ACG TGA GCT TCT TCG GAG TA; antisense: GGT CTG TCC CTC TTG TTT TCA TCT ATT), and IL-1R2 mRNA (sense: AGG AAT ACA ACA TCA CTA GGA ATA TTG AAC; antisense: TCA GTC TTG ACC CCA ATG ATG CT). Cytokine mRNA levels were normalized to a pool of non-lesioned brains. Quantitative PCR investigating temporal changes were conducted using C57BL/6 mice with 1, 2, 4, 6, 12 and 24 h survival, including shams and non-lesioned controls (*n* = 10–12/group).

### In situ hybridization

Alkaline phosphatase (AP)-conjugated oligodeoxynucleotide probe sequences for IL-1β mRNA and GAPDH mRNA were used, as described by Clausen et al. [11, 12]. In addition, the following probes were synthesised: IL-1Ra mRNA (5′TGC CCC CGT GGA TGC CCA AGA ACA C) and IL-1α mRNA (probe 1: 5′GTG CTG ATC TGG GTT GGA TGG TCT CTT C; probe 2: 5′GTT TCT GGC AAC TCC TTC AGC AAC ACG G). Probe specificity was validated as detailed by Clausen et al. [10], with the inclusion of sections from IL-1Ra-KO mice (Figure S1). Temporal profiles for IL-1Ra mRNA, IL-1α mRNA and IL-1β mRNA were obtained from one series of sections from C57BL/6 mice with 30 min, and 1, 4, 6, 12 and 24 h survival, including sections from non-lesioned controls (*n* = 6/group). IL-1Ra mRNA was also analyzed in one series of sections from mice with 5 days of survival (*n* = 10/group).

### Flow cytometry

Flow cytometry was performed using the FACSCalibur flow cytometer and CellQuest Pro Software (BD Bioscience) [10, 40]. IL-1α^+^ and IL-1β^+^ microglia [CD11b^+^CD45^dim^] and leukocytes [CD11^+^CD45^high^] were identified as detailed by Clausen et al. [10]. Samples analyzed for IL-1Ra protein were fixed and permeabilized in Cytofix/Cytoperm for 20 min at 4 °C. Cells were washed in 1 ml 1 × PermWash™ buffer (BD Bioscience), blocked using 10 % fetal calf serum (FCS) with 50 μg/ml Syrian Hamster IgG (11.3 mg/ml) (Jackson Immunoresearch) and incubated with anti-IL-1Ra prior to surface marker labeling of microglia and macrophages in mice with 6, 12 and 24 h survival in addition to non-lesioned controls (Ctl) (*n* = 4–8/group) [10]. BM cells were quantified based on the CFSE signal, and cell numbers were estimated in mice with 6 h survival as described previously [3, 64]. All antibodies used are listed in Table S3.

### Immunohistochemistry

*Mouse brain tissue* CD41, IL-1α and IL-1RII were visualized using the streptavidin/horseradish peroxidase (HRP) technique, IL-1β, using the peroxidase labeled “Ready to use” EnVision^+^ polymer (Dakocytomation, Denmark) [10, 12] and IL-1Ra and IL-1RI, using the Vectastain ABC kit (PK4005 and PK6104; Vector Laboratories) on series of sections from mice with 4, 6, 12 and 24 h survival in addition to non-lesioned controls (*n* = 6/group). *IHC double*-*fluorescencestaining* This was performed as detailed in Clausen et al. [10] on 16 sections spanning the anterior commissure including areas of both frontal and parietal cortices (*n* = 5–6 mice/staining). The specificity of staining for IL-1α, IL-1β and IL-1Ra was verified by the absence of staining in IL-1α/β-KO and IL-1Ra-KO brains, and the specificity of all other antibodies was verified by the substitution of the primary antibody with isotype or serum controls (Table S3), which were devoid of signal (data not shown). *Human brain tissue* Formalin-fixed paraffin-embedded [31] hematoxylin and eosin (HE)-stained tissue samples were evaluated by independent pathologists before serial sections were stained for IL-1Ra, rIgG2a, Iba1 CD45, CD68 and GFAP (Table S3). Slides were stained on the AutostainerPlus platform (Dako, Denmark) as previously described by Hermansen et al. [31]. The PowerVision^+^Poly_HRP IHC detection System (PV6107, Leica Biosystems) was used for the detection of all antibodies [31]. Sections stained with IgG or sections where primary antibodies were omitted were devoid of staining. *IHC double staining* was performed using the BenchMark fully automated staining instrument and the Vectastain Universal Elite ABC kit (Vector Labs, USA) [14]. Sections were counterstained with Mayer’s hematoxylin (Bie & Berntsen, Herlev, Denmark). All antibodies used are listed in Table S3.

### Scoring of IL-1Ra staining intensity

The infarcts were categorized according to age as follows at 1–2 days (*n* = 8) and 5 to ≥7 days (*n* = 13). The infarct core, the peri-infarct and normal-appearing tissue were identified in hematoxylin-stained sections. The regions were transferred onto parallel sections stained for IL-1Ra and scored as follows: Score 0: no staining; Score 1: very weak staining; Score 2: weak staining; Score 3: moderate staining; Score 4: strong staining; Score 5: very strong staining. The scoring was performed by two observers blinded to the age of the infarcts.

### Elisa

IL-1Ra (IL-1Ra Kit: MRA00, R&D) and IL-1β (IL-1β Kit: CMC0813, Invitrogen) ELISA were performed on one series of sections and conducted as specified in the ELISA kits. Measurements were normalized to the total protein content of the sample as measured using the method by Bradford [7].

### Western blotting

Western blotting was performed on one series of sections as described in Clausen et al. [9] with minor modifications. Western blotting for phosphorylated (p)-SAPK/JNK (Thr183/Tyr185) (1:1000), p-p44/p42 MAPK (ERK1/2)(1:2000) and p-p38 MAPK (Tyr180/Tyr182) (1:1000) (S9910 Kit, Cell Signaling) was performed using 4–12 % gels (Life Technologies) using transcription factor IIB (TFIIB) (Cell Signaling, 1:1000) as a loading control and SeeBlue Plus2 standard (Invitrogen) as an indicator of size. Bands were amplified using a SuperSignal^®^Extended duration substrate (34075, Thermo) and visualized by chemiluminescence using the FUSION 7X camera. Densitometry was performed using Image J (version 1.47v) (NIH) following recommendations of the Image J developers. Analysis was performed on two independent gels with *n* = 2/group and data were normalized to TFIIB and represented as percentages relative to naive LM mice.

### Statistical analysis

Quantitative data are presented as mean ± SD. Comparison between two groups was performed using Mann–Whitney or paired *t* test. Multiple comparisons were performed using one-way ANOVA and repeated-measures ANOVA, followed by appropriate post hoc tests. Correlations were established using the non-parametric Spearman test. Statistical analysis was performed using the Prism 5 software for Windows (GraphPad). Statistical significance was established for *P* < 0.05.

## Results

### Effect of IL-1Ra on infarct size

**Fig. 1:**
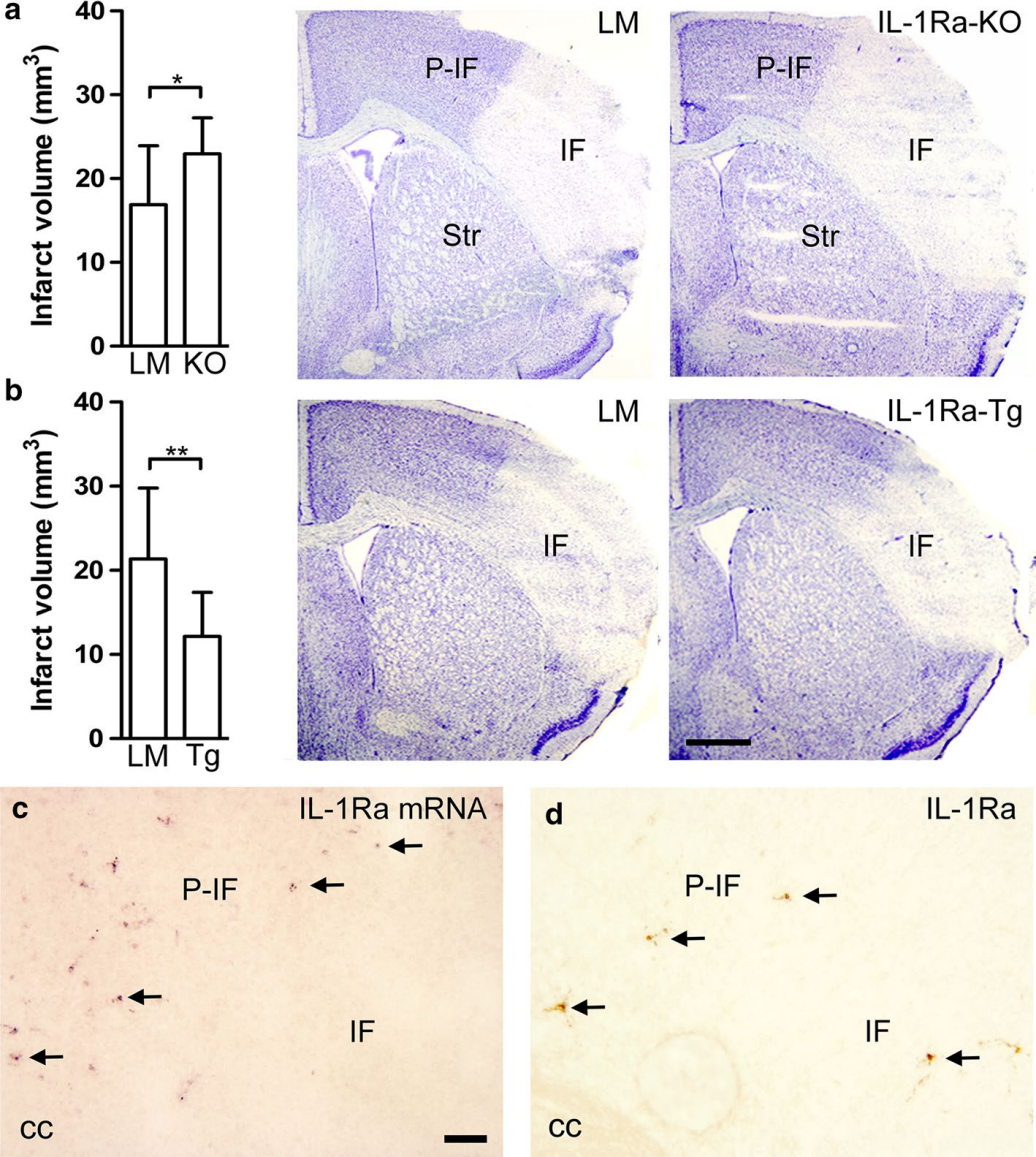
Endogenous IL-1Ra is neuroprotective. (**a**, **b**) Infarct volume estimated in LM and IL-1Ra-KO (KO) mice (**a**), and in LM  and IL-1Ra-Tg (Tg) mice (**b**), 24 h after pMCAo, with toluidine blue staining of the ischemic infarct, *n* = 14/group. Statistical data are presented as mean ± SD (Mann–Whitney test). *P < 0.05; **P < 0.01. **c**, **d** *ISH* showing IL-1Ra mRNA^+^ cells (*arrows* in **c**) and IHC showing IL-1Ra^+^ cells (*arrows* in **d**) in the peri-infarct 24 h after pMCAo. *cc* corpus callosum; *IF* infarct; *P-IF* peri-infarct; *Str* striatum. *Scale bars* 1 mm (**a**, **b**), and 100 µm (**c**, **d**)

To confirm the findings by others suggesting that endogenous IL-1Ra is neuroprotective in experimental stroke [43], we initially subjected IL-1Ra knockout (IL-1Ra-KO) mice, mice overproducing IL-1Ra under the control of the natural promoter (IL-1Ra-Tg) and their respective littermate (LM) mice to permanent middle cerebral artery (MCA) occlusion (pMCAo) (Table S1). Twenty-four hours after pMCAo, IL-1Ra-KO mice developed significantly larger infarcts (Fig. 1a) and IL-1Ra-Tg mice significantly smaller infarcts (Fig. 1b) than their respective LM controls. Additionally, at 24 h after pMCAo, we observed numerous IL-1Ra mRNA expressing (IL-1Ra mRNA^+^) cells, and IL-1Ra immunoreactive (IL-1Ra^+^) cells within the peri-infarct in C57BL/6 mice (Fig. 1c, d). The location of the IL-1Ra-producing cells in the tissue at risk for degeneration is consistent with a neuroprotective role of endogenous IL-1Ra.

### Cellular location of IL-1Ra and IL-1α/β mRNA and protein

**Fig. 2:**
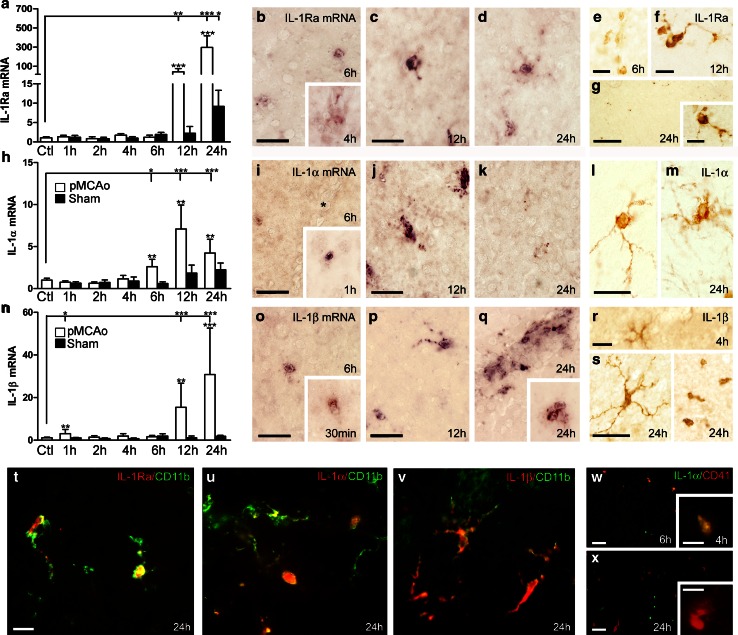
IL-1Ra and IL-1α/β synthesis is increased in CD11b^+^ cells in the peri-infarct after pMCAo. IL-1Ra (**a**–**g**), IL-1α (**h**–**m**), and IL-1β (**n**–**s**) mRNA and protein expression, and IHC double-fluorescence stainings (**t**–**x**) after pMCAo. **a**, **h**, **n** Quantitative PCR results showing temporal changes in IL-1Ra, IL-1α and IL-1β mRNA levels in pMCAo- and sham-operated mice, compared to non-lesioned controls (Ctl), *n* = 10–12/group. Statistical data are presented as mean ± SD (Kruskal–Wallis test with Dunns post hoc test). *P < 0.05; **P < 0.01; ***P < 0.001. **b**–**d**, **i**–**k**, **o**–**q** *ISH* showing IL-1Ra mRNA^+^ (**b**, **c**, **d**), IL-1α mRNA^+^ (**i**, **j**, **k**) and IL-1β mRNA^+^ (**o**, **p**, **q**) cells in the peri-infarct. By 24 h, IL-1β mRNA was expressed by single cells and vessel-associated cells (**q**). **e**–**g**, **l**, **m**, **r**, **s** IHC showing IL-1Ra^+^ cells (**e**–**g**), IL-1α^+^ cells and (**l**, **m**), IL-1β^+^ (**r**, **s**) cells in the peri-infarct. **t**–**v** IHC double-fluorescence staining showing co-localization of IL-1Ra (**t**), IL-1α (**u**) and IL-1β (**v**) to CD11b^+^ microglia 24 h after pMCAo. **w**, **x** IHC double-fluorescence staining showing co-localization of IL-1α to CD41^+^ platelets in the peri-infarct, 4 and 6 h (**w**), but not 24 h after pMCAo (**x**). *Scale bars*: 20 µm (**b**–**d**, **i**–**k**, **o**–**q**), 10 µm (**e**, **f**, **g**, **l**, **m**, **r**–**x **, and insert in **g**) and 100 µm (**g**)

To obtain detailed insight into the cellular orchestration of IL-1Ra and IL-1(α**/**β), we examined the synthesis of all three cytokines in C57BL/6 mice after pMCAo. By the use of quantitative reverse transcription PCR (qPCR), we observed small fluctuations in IL-1Ra mRNA until 6 h (Fig. 2a) and a significant up-regulation at 12 and 24 h after pMCAo, compared to non-lesioned and sham controls (Fig. 2a). Within the peri-infarct, we found multiple process-bearing microglial-like cells with abundant IL-1Ra mRNA (Fig. 2b–d) and protein accumulation (Fig. 2f, g) 6, 12 and 24 h after pMCAo, and round vessel-associated leukocyte-like cells from 4 to 6 h after pMCAo (insert in Fig. 2b, e).

The level of IL-1α mRNA was significantly up-regulated at 6, 12 and 24 h (Fig. 2h), with IL-1α mRNA and protein peaking in microglial-like cells located in the peri-infarct 12 and 24 h after pMCAo (Fig. 2i–m). IL-1β mRNA was increased at 1, 12 and 24 h (Fig. 2n) and showed larger increases than IL-1α mRNA (Fig. 2h). IL-1β mRNA^+^ cells were detected from 30 min after pMCAo and onward, increasing in number until 24 h, at which time IL-1β mRNA^+^ cells also occurred perivascularly (Fig. 2o–q). IL-1β was expressed by microglial-like cells in the peri-infarct at 4 and 24 h and by leukocyte-like cells 24 h after pMCAo (Fig. 2r, s). When performing Spearman correlation analysis, we observed a positive correlation between IL-1Ra and IL-1β mRNA at 1, 2, 6, 12 and 24 h after pMCAo and between IL-1Ra and IL-1α mRNA 24 h after pMCAo (Table S4). No correlation was observed between IL-1α and IL-1β mRNA (Table S4). IHC double-fluorescence staining showed the expression of IL-1Ra, IL-1α and IL-1β in CD11b^+^ cells (Fig. 2t–v), but not in glial fibrillary acidic protein-expressing astrocytes (data not shown), suggesting microglia and/or leukocytes as the major source of these cytokines. In addition, we found IL-1α in CD41^+^ platelets at 4–6 h, but not 24 h, after pMCAo (Fig. 2w, x), which adds to in vitro findings of IL-1α in platelets [62].

Since IL-1(α**/**β) and IL-1Ra are known to impact on infarct development, we additionally analyzed the mRNA expression of IL-1R1 and -1R2 by qPCR. We found small fluctuations in IL-R1 mRNA until 6 h and small increases at 12 and 24 h after pMCAo compared to non-lesioned and sham controls (Figure S2a, c). IL-1R1 immunoreactivity was associated with vascular cells at 6 h and glial-like cells at 24 h after pMCAo (Figure S2b). In comparison, IL-1R2 mRNA was significantly increased at 6, 12 and 24 h compared to non-lesioned and sham controls (Figure S2c), significantly correlating to the ischemia-induced increase in IL-1α mRNA (Table S4) and the occurrence of IL-1R2^+^ leukocyte-like cells in the peri-infarct (Figure S2d).

### IL-1Ra is predominantly expressed by microglia

**Fig. 3:**
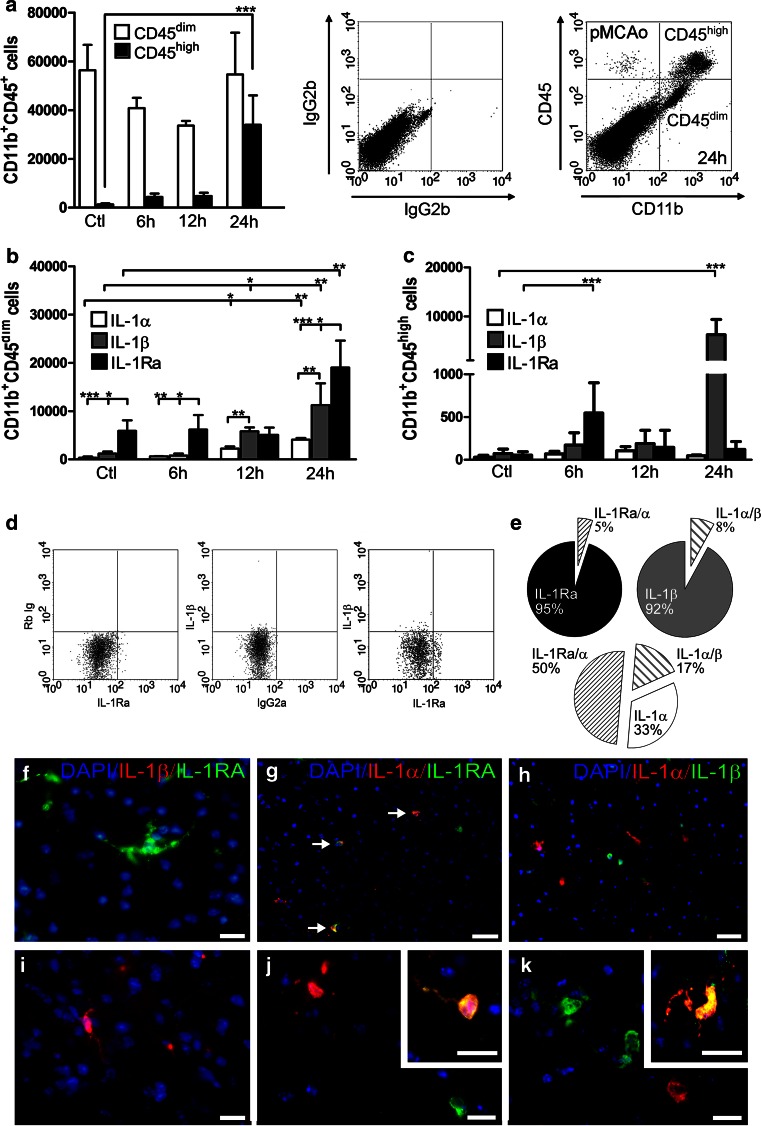
IL-1Ra, IL-1α and IL-1β are expressed by largely different microglial subsets. **a** Flow cytometric quantification of CD11b^+^CD45^dim^ microglia and CD11b^+^CD45^high^ leukocytes in non-lesioned C56BL/6 controls (Ctl) and in C56BL/6 mice 6, 12 and 24 h after pMCAo (*left*), *n* = 4–8/group, with *dot plots* (*right*) showing flow cytometric profiles and the isotype quadrant from a mouse with 24 h survival. **b**, **c** Flow cytometric quantification of IL-1Ra^+^, IL-1α^+^, and IL-1β^+^ CD11b^+^CD45^dim^ microglia (**b**) and CD11b^+^CD45^high^ leukocytes (**c**), *n* = 4/group. Statistical data are presented as mean ± SD (Kruskal–Wallis test with Dunns post hoc test). *P < 0.05, **P < 0.01, ***P < 0.001. **d** *Dot plot* (*left*) with isotype quadrants showing gated CD11b^+^CD45^dim^ microglia expressing either IL-1Ra or IL-1β 24 h after pMCAo. **e** *Pie charts* show the percentage of gated CD11b^+^CD45^dim^ microglia co-expressing either IL-1Ra and IL-1α or IL-1β and IL-1α, with no co-expression of IL-1Ra and IL-1β 24 h after pMCAo (co-expression is indicated as percentages outside the pie charts). **f**–**k** IHC double-fluorescence staining showing IL-1Ra and IL-1β (**f**, **i**), IL-1Ra and IL-1α (**g**, **j**), and IL-1β and IL-1α (**h**, **k**) in the peri-infarct 24 h after pMCAo. IL-1Ra and IL-1β are expressed in spatially segregated microglia (**f**, **i**, from the same section). *Scale bars* 10 µm (**f**, **i**), 50 µm (**g**, **h**), 10 µm (**j**, **k**), and 10 µm (*inserts*)

A quantitative assessment of microglial versus leukocyte-derived IL-1Ra, IL-1α and IL-1β was performed by flow cytometry separating CD11b^+^ cells into CD11b^+^CD45^dim^ microglia and CD11b^+^CD45^high^ leukocytes (Fig. 3a) [10, 40, 58]. As previously shown [10, 40], the number of recruited CD11b^+^CD45^high^ leukocytes gradually increased from 6 to 24 h after pMCAo in C57BL/6 mice, whereas the number of CD11b^+^CD45^dim^ microglia overall remained constant (Fig. 3a). IL-1Ra, IL-1α and IL-1β were expressed by both CD11b^+^CD45^dim^ microglia (Fig. 3b) and CD11b^+^CD45^high^ leukocytes (Fig. 3c), however, with significant differences in baseline and temporal expression profiles (Figs. 3b, S3a–c). Interestingly, there were significantly higher numbers of IL-1Ra^+^ compared to IL-1α^+^ and IL-1β^+^ microglia in non-lesioned control mice and at 6 h after pMCAo, but not at 12 h, when both IL-1α^+^ and IL-1β^+^ microglia had increased compared to controls (Fig. 3b). By 24 h, the proportion of IL-1Ra^+^ microglia reached 34 ± 11 % [mean ± SD, (*n* = 4)] of the gated CD11b^+^CD45^dim^ population, while IL-1α^+^ reached 9 ± 1 % [mean ± SD, (*n* = 4)] and IL-1β^+^ reached 18 ± 5 % [mean ± SD, (*n* = 4)].

The number of IL-1β^+^ microglia exceeded the number of IL-1α^+^ microglia after pMCAo at all times of observation (Fig. 3b). Surprisingly, few CD11b^+^CD45^high^ leukocytes were IL-1Ra^+^ apart from 6 h after pMCAo, when 16 ± 7 % (*n* = 4) were IL-1Ra^+^ (Fig. 3c). By 24 h, less than 0.3 ± 0.05 % (*n* = 4) of the leukocytes were either IL-1Ra^+^ or IL-1α^+^, whereas 17 ± 9 % (*n* = 4) expressed IL-1β (Fig. 3c). A comparison of the number of microglia and leukocytes expressing IL-1Ra, IL-1α and IL-1β showed that microglia was the major source of IL-1Ra and IL-1α, whereas both microglia and leukocytes were a source for IL-1β 24 h after pMCAo (Fig. 3b, c; Table S5). These findings were confirmed by the use of GFP irradiation BM chimeric mice (Figure S3a–c) showing that the majority of the IL-1Ra^+^ and IL-1α^+^ cells were GFP^−^ microglia (Figure S3a, b), while IL-1β was expressed by both GFP^−^ microglia and GFP^+^ leukocytes (Figure S3c). These results clearly show microglia as the major source of neuroprotective IL-1Ra after pMCAo, and additionally suggest that increasing IL-1Ra production in recruited cells could be of relevance in stroke therapy.

### IL-1Ra and IL-1β are expressed by segregated subsets of microglia

Based on our previous findings showing that IL-1β and TNF are expressed by largely different subsets of microglia [10], we assessed the proportion of CD11b^+^CD45^dim^ microglia co-expressing IL-1Ra, IL-1α and/or IL-1β 24 h after pMCAo. We found IL-Ra and IL-1β to be produced by segregated subsets of CD11b^+^CD45^dim^ microglia with no co-expression (Fig. 3d, e). Approximately, 5 % of the gated IL-1Ra^+^ microglia co-expressed IL-1α (5.1 ± 1.9 % (*n* = 4)), while significantly higher proportions, approximately 50 % of the gated IL-1α^+^ cells co-expressed IL-1Ra (50.4 ± 6.5 % (*n* = 4)) (Figure S3d). Further, we found that approximately 8 % of the gated IL-1β^+^ microglia co-expressed IL-1α (8.0 ± 4.1 % (*n* = 4)) (Fig. 3e), with higher proportions, and approximately 17 % of the gated IL-1α^+^ microglia co-expressing IL-1β (17.3 ± 6.1 % (*n* = 4)) (Figs. 3e, S3d). Double-immunofluorescence staining confirmed the absence of co-expression between IL-1Ra and IL-1β 24 h after pMCAo, showing spatially segregated cells producing either IL-1Ra or IL-1β (Fig. 3f, i). We confirmed the co-expression of IL-1Ra and IL-1α (Fig. 3g, j) and IL-1α and IL-1β; the latter however was only observed in a few number of cells 24 h after pMCAo (Fig. 3h, k). Taken together, these results demonstrate a functional diversity among microglia at the site of injury.

### Increasing IL-1Ra production in infiltrating leukocytes can decrease infarct size

**Fig. 4:**
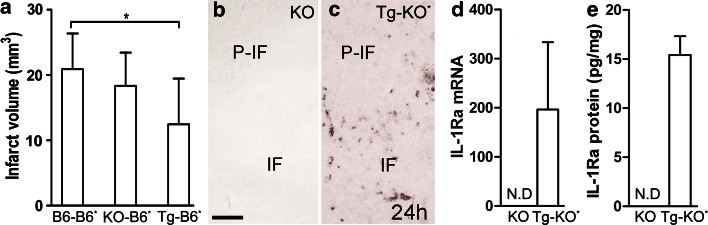
Leukocyte-derived IL-1Ra reduces ischemic infarction in IL-1Ra-B6* BM chimeric mice. **a** Infarct volumes estimated in whole body-irradiated* C57BL/6 (B6*) mice reconstituted with BM from either B6, IL-1Ra-KO, or IL-1Ra-Tg mice, 24 h after pMCAo, *n* = 12–14/group. Statistical data are presented as mean ± SD (Kruskal–Wallis test with Dunns post hoc test), *P < 0.05. **b**, **c** *ISH* for IL-1Ra mRNA of sections from IL-1Ra-KO mice showing absence of IL-1Ra mRNA^+^ (**b**), and of irradiated IL-1Ra-KO mice (KO*) reconstituted with BM from IL-1Ra-Tg mice, showing the presence of infiltrating IL-1Ra mRNA^+^ cells in the peri-infarct, 24 h after pMCAo (**c**). **d**, **e** IL-1Ra mRNA and IL-1Ra protein levels in the same groups of mice as illustrated in (**b**, **c**), and determined by qPCR (**d**) and ELISA (**e**). *IF* infarct; *ND* none detected; *P-IF* peri-infarct. *Scale bar* 100 µm

Based on the observation that IL-1Ra is expressed by a small subset of infiltrating CD11b^+^CD45^high^ leukocytes, we next tested whether increasing IL-1Ra production in infiltrating leukocytes would be neuroprotective. As previously done [10, 40], we used a BM chimeric approach, comparing infarct development in whole body-irradiated recipient C57BL/6 mice (B6*) mice reconstituted with BM cells from either IL-1Ra-Tg, IL-1Ra-KO or B6 mice (Table S1). We observed no difference in infarct size between KO-B6* BM chimeric mice and B6–B6* BM chimeric mice (Fig. 4a), which indicates that leukocytes under normal conditions fail to produce sufficient IL-1Ra to influence infarct development. In comparison, the Tg–B6* BM chimeras developed significantly smaller infarcts compared to the B6–B6* BM chimeric mice, 24 h after pMCAo (Fig. 4a), suggesting that increasing the level of leukocyte-produced IL-1Ra is neuroprotective.

To track IL-1Ra synthesizing BM-derived leukocytes in the ischemic brain, we also subjected IL-1Ra-KO mice reconstituted with BM cells from IL-1Ra-Tg mice (Tg–KO*) to pMCAo. *ISH* of sections from these mice showed multiple IL-1Ra mRNA^+^ cells in the peri-infarct of irradiated IL-1Ra-KO mice reconstituted with BM cells from IL-1Ra-Tg mice (Tg–KO*), compared to no cells in IL-1Ra-KO mice, 24 h after pMCAo (Fig. 4b, c). By analyzing RNA and protein isolated from parallel sections, we, as expected, observed a robust expression of IL-1Ra mRNA and protein in Tg–KO* mice, and not in IL-1Ra-KO mice, 24 h after pMCAo (Fig. 4d, e). Interestingly, the increase in IL-1Ra synthesis was associated with a lower ischemia-induced increase in IL-1β mRNA levels in Tg–KO* compared to IL-1Ra-KO mice (qPCR, IL-1Ra-KO: 96.6 ± 67.5, *n* = 12; Tg–KO*: 16.6 ± 11.3, *n* = 14; *P* < 0.001; Mann–Whitney test).

In combination, these results provide first evidence that increasing IL-1Ra expression by infiltrating leukocytes, controlled by endogenous kinetics can promote and support neuroprotective signaling pathways in the ischemic cortex.

### IL-1Ra-overproducing BM cells infiltrate the ischemic cortex and induce IL-1Ra synthesis in microglia

**Fig. 5:**
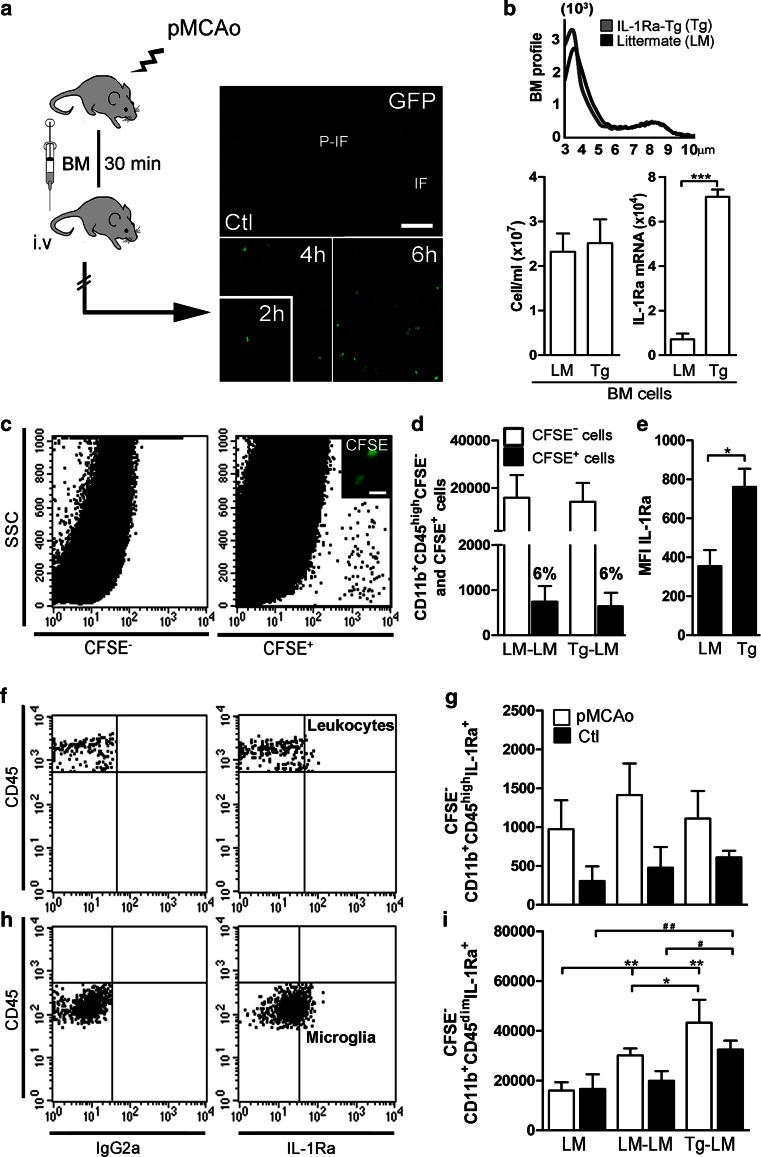
IL-1Ra-overproducing BM cells stimulate microglial production of IL-1Ra. **a** GFP^+^ BM cells in the cortex of C57BL/6 mice 2, 4 and 6 h after pMCAo, but not in non-lesioned controls (Ctl). **b** BM profiles, cells numbers and IL-1Ra mRNA expression in BM from IL-1Ra-Tg (Tg) and LM mice. **c** *Dot plot*s showing flow cytometric profiles of CFSE^+^ BM cells 6 h after pMCAo (5.5 h after BM transplantation). **d** Infiltrating CFSE^+^ BM cells and CFSE^−^ CD11b^+^CD45^high^ leukocytes in LM–LM and Tg–LM mice 6 h after pMCAo. **e** MFI of IL-1Ra in gated CFSE^+^ BM cells 6 h after pMCAo, the CFSE^+^ BM cells originated from LM and IL-1Ra-Tg (Tg) donor mice. **f**–**i** *Dot plots* showing flow cytometric profiles with isotype quadrants (**f**, **h**) and quantification of IL-1Ra^+^ CD11b^+^CD45^high^CFSE^−^ leukocytes (**g**) and IL-1Ra^+^ CD11b^+^CD45^dim^CFSE^−^ microglia (**i**) in LM, LM–LM and Tg–LM 6 h after pMCAo. Statistical data are presented as mean ± SD, *n* = 4/group (Kruskal–Wallis test with Dunns post hoc test), *^#^ *P* < 0.05, **^##^ *P* < 0.01. *Scale bar* 100 µm (**a**) and 10 µm (**c**)

Having shown that leukocyte-derived IL-1Ra can be neuroprotective using irradiation BM chimeric mice, we next tested the ability of BM cells to infiltrate the ischemic cortex when injected post-stroke. First, we evaluated the ability of BM cells to infiltrate the developing infarct, by injecting GFP^+^ BM cells into the tail vein of C57BL/6 mice 30 min after pMCAo. We identified GFP^+^ cells as soon as 2 h after pMCAo, increasing in numbers at 4 and 6 h (*n* = 4/group), while no cells were found in the cortex of non-lesioned controls (Fig. 5a). Next, we analyzed BM samples isolated from IL-1Ra-Tg and LM mice, and showed that samples, as expected, were similar regarding the BM profiles and the unit volume (cells/ml), but that BM cells from IL-1Ra-Tg mice showed higher expression of IL-1Ra mRNA than BM cells from sibling donors (Fig. 5b).

Prior to the therapeutic injections of BM cells obtained from either IL-1Ra-Tg or LM mice, we labeled them with the fluorescent marker CFSE (carboxyfluorescein diacetate succinimidyl ester), allowing the cellular detection by flow cytometry 6 h after pMCAo (Fig. 5c). We found that approximately 6 % of the recruited CD11b^+^CD45^high^ cells in both LM–LM and Tg–LM mice were CFSE^+^ BM cells, and that the numbers of recruited CFSE^−^ CD11b^+^CD45^high^ leukocytes were comparable 6 h after pMCAo (Fig. 5d). We analyzed the mean fluorescence intensity (MFI) of IL-1Ra in the CFSE^+^ BM recruits uncovering significantly higher IL-1Ra synthesis in BM cells originating from IL-1Ra-Tg (Tg) compared to LM mice 6 h after pMCAo (Fig. 5e).

Because the major source of IL-1Ra in the ischemic brain is microglia, supplemented by few recruited leukocytes (Fig. 3b, c), we asked whether the CFSE^+^ BM recruits could impact IL-1Ra synthesis by these cells. Flow cytometry analysis showed that the number of IL-1Ra^+^CD11b^+^CD45^high^ leukocytes was comparable in Tg–LM, LM–LM and LM mice 6 h after pMCAo, as well as in their non-lesioned control mice (Fig. 5f, g). In contrast, we observed a significant increase in the number of IL-1Ra^+^CD11b^+^CD45^dim^ microglia in both Tg–LM and LM–LM mice compared to non-treated LM mice 6 h after pMCAo (Fig. 5h, i). The finding of significantly higher numbers of IL-1Ra^+^CD11b^+^CD45^dim^ microglia in Tg–LM compared to LM–LM mice stressed the source-benefit of the cells, revealing BM from IL-1Ra-Tg mice as potent inducers of IL-1Ra synthesis by microglia after stroke (Fig. 5h, i). These data were supported by ELISA data showing significantly more IL-1Ra protein in Tg–LM mice compared to LM mice 6 h after pMCAo (Tg–LM: 10.7 ± 6.5, LM–LM: 2.7 ± 2.6; LM: 2.7 ± 3.2 pg/mg; *n* = 5 mice/group). Thus, microglia are highly plastic cells which can be induced to increase their production of neuroprotective IL-1Ra.

Since mitogen-activated protein kinase (MAPK) signaling pathways play an important role in the early inflammatory response after pMCAo (25), the effect of the BM treatment on MAPKs signaling pathways, including extracellular signal-regulated kinase (ERK), Jun N-terminal kinase (JNK) as well as p38, was explored in the tissue from the same mice (Figure S4a–c). The level of phosphorylated (p) JNK was elevated by 60–80 % 6 h after pMCAo in mice treated with IL-1Ra-overproducing BM cells, compared to pMCAo LM mice (Figure S4b). These findings suggest that the increased availability of IL-1Ra in the BM-treated mice modulate the ischemia-induced activation of the JNK pathway.

### IL-1Ra-producing BM cells are neuroprotective, reduce brain IL-1β levels and improve functional outcome

**Fig. 6:**
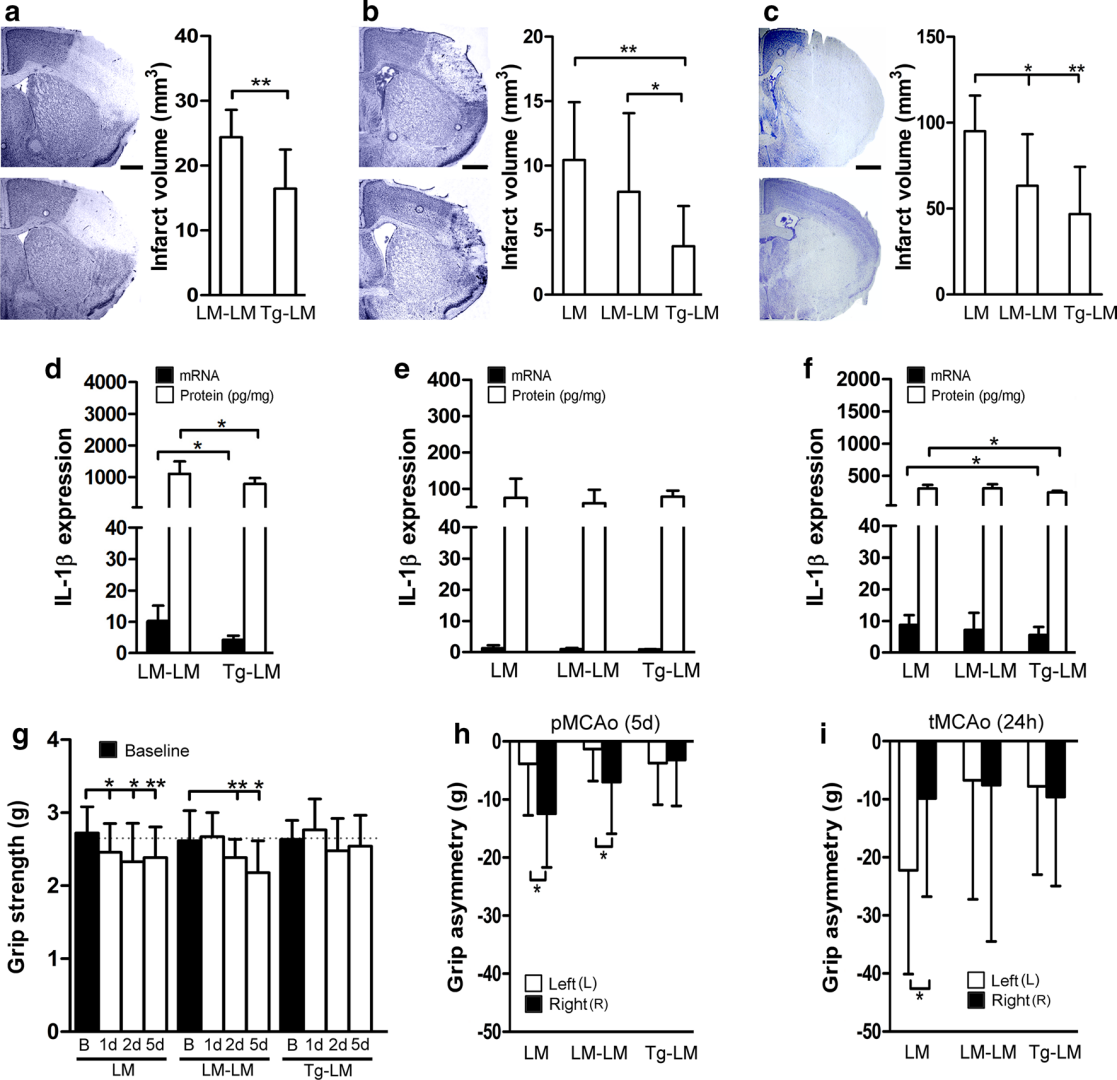
IL-1Ra-overproducing BM cells have a therapeutic effect after both pMCAo and tMCAo. **a**–**c** Toluidine *blue* stainings and infarct volume estimation 24 h after pMCAo, *n* = 14/group (**a**), 5 days after pMCAo, *n* = 14/group (**b**), and 24 h after tMCAo, *n* = 12–17/group (**c**), with infarct volume estimation shown for LM, LM–LM and Tg–LM mice. **d**–**f** Quantitative PCR and ELISA performed on sections parallel to those used in (**a**–**c**) show differences in IL-1β mRNA and protein among the treatment groups. **g**, **h** Grip strength of the affected right front paws (**g**) and grip strength asymmetry in right versus left front paw (**h**) in LM, LM–LM and Tg–LM mice 1, 2 and 5 days after pMCAo compared to baseline. The *stippled line* (**g**) indicates the grip strength of the left front paw. **i** Grip strength asymmetry in right versus left front paw in LM, LM–LM and Tg–LM mice 1 day after tMCAo. Statistical data are presented as mean ± SD. (Mann–Whitney test (**a**, **d**), Kruskal–Wallis test with Dunns post hoc test (**b**, **c**, **e**, **f**), repeated-measures ANOVA with Dunnett post hoc test (**g**) or Wilcoxon matched pairs test (**h**, **i**). *P < 0.05; **P < 0.01. *B* baseline. *Scale bars* 1 mm

As an increase in IL-1Ra production by BM cells or microglia could impact the infarct development, thereby changing the neurological outcome, we finally investigated the therapeutic effect of IL-1Ra-overproducing BM cells in mice subjected to either pMCAo or tMCAo (Fig. 6). BM cells harvested from either LM mice or IL-1Ra-Tg mice (Tg) were injected into the tail veins of LM recipients 30 min after MCAo (Table S1). We first investigated the effect of IL-1Ra-overproducing BM cells in mice subjected to pMCAo with 24 h or 5 days survival (Table S1). We observed a significant reduction in infarct size in Tg–LM compared to LM–LM mice 24 h after pMCAo (survival rate/group >95 %) (Fig. 6a). LM–LM mice showed similar infarct sizes as non-treated LM mice 24 h after pMCAo (compared to Fig. 1b). Encouraged by the prominent effect of IL-1Ra^+^ BM cells, we allowed mice to survive for 5 days to assess any physiological and/or behavioral improvements of this treatment strategy. By day 5, we observed a significant reduction in infarct volume in Tg–LM mice compared to LM–LM mice (survival rate/group >95 %), with no difference between LM–LM-treated mice and LM mice (Fig. 6b). The protective effect of IL-1Ra was not a result of decreased body temperature, as rectal temperature recordings (up to 3 h) showed no differences between treatment groups (Figure S5a). Additionally, blood gas parameters (pO_2_/pCO_2_, pH, electrolytes, glucose/lactate) recorded 30 min after BM injections (i.e., 1 h after pMCAo) showed no difference among various experimental groups (LM, LM–LM and Tg–LM mice) or when compared to non-lesioned mice (Figure S5b-i*).* When performing in situ hybridization, we observed IL-1Ra mRNA^+^ cells in the peri-infarct in none of the ten LM mice, one out of ten LM–LM and in two out of ten Tg–LM mice 5 days after pMCAo (Figure S6a, shown for Tg–LM). These results matched qPCR results obtained on RNA isolated from parallel sections, showing elevated levels of IL-1Ra mRNA in Tg–LM compared to both LM–LM and LM 5 days after pMCAo (Figure S6b). These results suggest the continued presence of infiltrating IL-1Ra mRNA^+^ BM cells, and/or a prolonged expression of IL-1Ra mRNA by host cells in BM-grafted mice, 5 days after pMCAo.

We next tested the neuroprotective effect of IL-1Ra-overproducing BM cells, in the model of tMCAo (Table S1), which gives rise to a large lesion comprising both cortical and subcortical areas (Fig. 6c). We observed a significant reduction in infarct size in Tg–LM mice and LM–LM mice compared to LM controls, 24 h after tMCAo (Fig. 6c) (survival rate/group >95 %), thereby supporting the findings in mice subjected to pMCAo, additionally showing an infarct-reducing effect of LM BM cells 24 h after tMCAo.

To address a possible mechanism responsible for the beneficial effect of increased IL-1Ra, we quantified IL-1β mRNA and protein in parallel sections by qPCR and ELISA (Fig. 6d–f). The ischemia-induced increase in IL-1β mRNA was significantly smaller in Tg–LM mice compared to LM–LM mice 24 h after pMCAo (Fig. 6d). Consistent with this observation, we also observed less IL-1β protein in Tg–LM mice compared to LM–LM mice 24 h after pMCAo (Fig. 6d). By day 5, both IL-1β mRNA and protein in LM, LM–LM and Tg–LM were back at baseline levels (Fig. 6e), as measured in non-lesioned B6 mice (see Fig. 2c for mRNA) and non-lesioned LM mice [IL-1β: 137 ± 72 pg/mg (mean ± SD), *n* = 3]. Similar to the pMCAo mice, we also observed a smaller ischemia-induced increase in IL-1β mRNA and protein in Tg–LM mice compared to LM mice 24 h after tMCAo (Fig. 6f), collectively suggesting an effect of increased IL-1Ra production on IL-1β expression after both pMCAo and tMCAo.

We used a battery of behavioral tests to assess the functional recovery in mice subjected to 5 days pMCAo or mice subjected to tMCAo. As the MCA supplies cortical areas controlling the contralateral front and hind limb, we measured the neuromuscular function of the left and right forelimbs (Fig. 6g–i). We detected a significant contralateral weakness in the right (R) front paw of LM mice 1, 2 and 5 days after pMCAo compared to normal baseline strength (Fig. 6g). A weakness first observed on day 2 and 5 after pMCAo in LM–LM mice was accordingly not observed in Tg–LM mice (Fig. 6g). By day 5, both LM and LM–LM mice showed pronounced grip asymmetry in the left and right forelimbs, whereas the performance of Tg–LM mice was comparable in the two forelimbs (Fig. 6h). A similar grip strength outcome was observed in LM–LM and Tg–LM mice 24 h after tMCAo (Fig. 6i). In addition, performing the Hargreaves and open field test on mice subjected to tMCAo, we observed an improved functional recovery in Tg–LM-treated mice (Table S6). Hargreaves test revealed similar nociception in the left and right hind paw prior to surgery with LM and LM–LM mice showing a significantly lower sensitivity compared to Tg–LM mice 24 h after tMCAo (Table S6). Using the open field test, we observed that the Tg–LM mice exhibited a more explorative activity, revealing that LM–LM and Tg–LM mice traveled longer (cm) and faster (cm/s) than LM mice 24 h after tMCAo. However, only Tg–LM mice showed reduced anxiety-related behavior spending more time in the center of the maze (Table S7).

Unlike LM and LM–LM mice, the Tg–LM pMCAo mice did not succumb to a significant 3 % weight loss (*P* < 0.01) on day 5, suggesting improved feed efficiency and physical well-being of the mice (weight loss/group after 5 days: LM 3.7 %; LM–LM 3.2 %; Tg–LM 0.2 %) (Figure S5j).

### IL-1Ra in human stroke patients

**Fig. 7:**
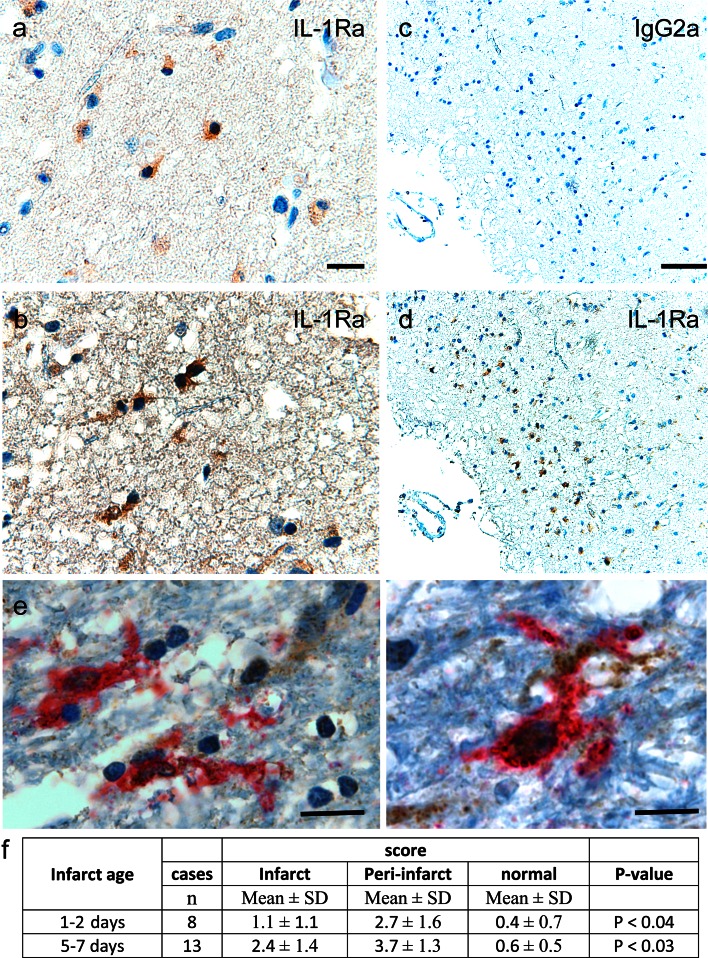
IL-1Ra production in the human stroke brain. **a**–**d** IHC showing IL-1Ra^+^ cells in the peri-infarct (**a**) and infarct core (**b**), 24 h after stroke onset. **c**, **d** IHC staining of parallel sections showing the IgG2a isotype control (**c**) and IL-Ra^+^ cells (**d**). **e** IHC Double staining showing co-localization of IL-1Ra to CD45^+^ microglial-like cells in the peri-infarct tissue 24 h post-stroke. **f** IHC score of the intensity of the IL-1Ra staining in infarct, peri-infarct and normal-appearing brain tissue. *Scale bars* 10 µm (**a**, **b**, **e**), and 50 µm (**c**, **d**)

Despite evidence that IL-1Ra is an important neuroprotective cytokine, its presence in the human brain after a stroke has not yet been investigated. Here, we show that IL-1Ra^+^ cells are present both in the peri-infarct (Fig. 7a) and the infarct core (Fig. 7b) 24 h after stroke onset (Fig. 7a–e). Scoring of IL-1Ra expression in three distinct regions, e.g., infarct core, peri-infarct and normal-appearing tissue, showed higher IL-1Ra expression in the peri-infarct compared to infarct core both 1–2 days (*P* < 0.04) and 5 to ≥7 days (*P* < 0.03) post-stroke (Fig. 7f). At ≥7 days, there was a marked increase of IL-1Ra^+^ cells (Table S2). Regions of infarct core, peri-infarct and normal-appearing tissue were identified based on parallel series of HE-stained sections. Iba1, CD45, CD68 and GFAP were used to distinguish the topography of IL-1Ra^+^ cells (Figure S7). IHC double staining showed IL-1Ra and CD45 co-expressing microglia 24 h post-stroke (Fig. 7e). These results provide novel insight into the production of IL-1Ra in the human brain and emphasize the translational relevance of our experimental results.

## Discussion

**Fig. 8:**
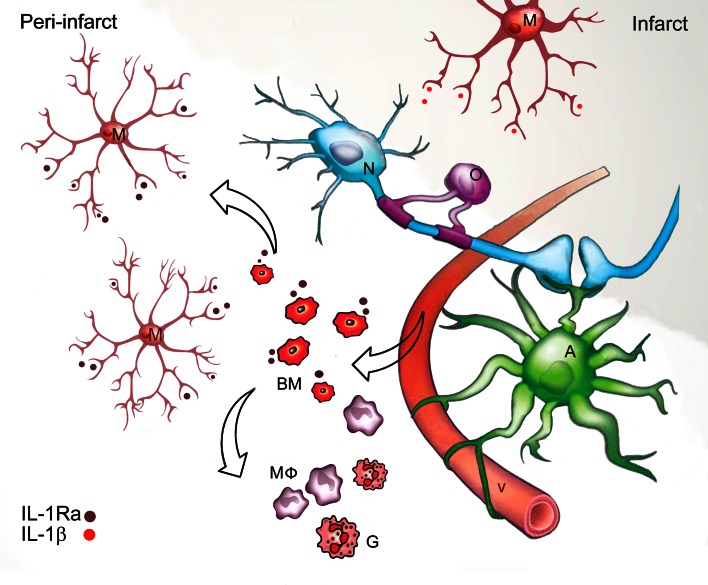
Schematic illustration of the presumed BM cell and microglial cross talk in the peri-infarct. The intravenously administered BM cells infiltrate the neural parenchyma in the peri-infarct where they ensure neuroprotection by interfering with the IL-1 self-promoting cycle, thereby stimulating subsets of microglia to secrete IL-1Ra. Infiltrating monocyte-derived macrophages and granulocytes produce insignificant amounts of IL-1Ra; however, macrophages are a major source of IL-1β. Note the heterogeneity of the microglia with some producing IL-1Ra and others producing IL-1β. *A* astrocyte; *G*: granulocytes; *M*: microglia; *MФ* macrophages; *N* neuron; *O*: oligodendrocyte; *v* vessel. Modified with permission from Dr. Ben Barres [2]

The present study shows a novel function of therapeutically injected IL-1Ra-producing BM cells in stroke, as these cells enhance the production of anti-inflammatory IL-1Ra by microglia, collectively protecting the peri-infarct as demonstrated by reduced infarct volumes and improved functional outcome in mice. We show that IL-1Ra and IL-1β are expressed by segregated subsets of microglia, which, coupled with similar findings for TNF and IL-1β [10], supports the view of a functional heterogeneity among microglia [17, 18, 30, 51, 53]. As microglia react dynamically to changes, with input and feedback signals arising from cells within the peri-infarct which most likely affects their functional state, we suggest that microglial activation is not an “all-or-none” process in the brain. Antagonizing IL-1 is neuroprotective and our findings suggest that BM cells encoding the secreted form of (s)IL-1Ra under the control of its endogenous promoter may ensure neuroprotection by abrogating microglial/macrophage activation by interfering in the IL-1 self-promoting cycle toward neuroinflammation [20] (Fig. 8). Surprisingly, little is known about the production of IL-1Ra in situ and its neuroprotective role in the stroke-injured brain [43]. Although, a recent cross-laboratory study suggests that post-stroke peripheral administration of IL-1Ra is neuroprotective in mice [44], pharmacokinetic studies in patients have shown that rIL-1Ra crosses the blood brain barrier very slowly [28] and has a short half-life in the circulation [26], the challenge being to achieve therapeutic concentrations within the shortest time possible [24]. Although challenging to administer, the strength of a cell-based therapy is the ability of cells to actively infiltrate the neural parenchyma and modulate local inflammatory responses and neuronal survival in the tissue at risk over time.

In this study, we also report on the changes in the cellular IL-1(α/β) content, identifying both microglia and platelets as the source of IL-1α and microglia and leukocytes as the source of IL-1β after pMCAo. Our data contradicts findings arguing that IL-1α is the predominant form of IL-1 following stroke [44], but supports the view that IL-1α plays an important role in stroke pathology, by strengthening in vitro findings of IL-1α in platelets [62]. The presence of IL-1α-carrying platelets likely affects the balance between IL-1/IL-1Ra in the infarcted cortex. By 24 h, we observed a few IL-1(α/β) co-producing microglia, expected to be linked to IL-1α secretion as IL-1β has been identified as a functional shuttle for IL-1α release [23]. In parallel, we observed multiple IL-1α/IL-1Ra co-producing microglia supporting the view that intracellular IL-1Ra could regulate the action of intracellular IL-1α [47]. Overall, these results can explain the shift toward early IL-1-induced neuroinflammation and illustrate the complexity of cellular cross talk following stroke. Imbalance between IL-1 and IL-1Ra has been shown to determine the degree of cardiac remodeling after myocardial infarction [1], and a high IL-1Ra/IL-1β ratio is associated with a better outcome in patients after traumatic brain injury [34].

Microglia and not infiltrating leukocytes attenuate the effect of IL-1 in stroke pathology, serving as an endogenous pool of IL-1Ra, as also suggested from our analysis of autopsy material from stroke patients. Our results demonstrate that microglia become effective on demand, by increasing their IL-1Ra production when encountering IL-1Ra-producing BM cells, leading to attenuation of IL-1-induced inflammation and neurodegeneration. However, to substantiate this treatment approach as neuroprotective, studies will have to be performed in female mice, aged mice and in mice with post-stroke end points greater than 5 days. Furthermore, to be of therapeutic value in the treatment of acute stroke, this treatment strategy shall also be proven to be beneficial using prolonged therapeutic time windows and ideally enhance the outcome when and if combined with thrombolysis therapy.

We suggest that BM cells, through the release of IL-1Ra, promote a neuroprotective microglial phenotype in the stroke-injured cortex, a view which is supported by evidence showing that mesenchymal stem cells can shape microglial effector function in vitro and promote a neuroprotective microglial phenotype [25]. We further provide insights into the functional effect of IL-1Ra-producing BM cells by showing their modulation of JNK/SAPK signaling, a key regulatory pathway for neurite outgrowth [55], neuronal differentiation and plasticity [63] and cell survival [16]. As mesenchymal stem cells are a recognized source of IL-1Ra [50], our finding that BM cells modulate microglial IL-1Ra production after stroke in mice may also shed light on an unresolved mechanism underlying the efficacy of mesenchymal stem cell therapy in stroke [29, 37].

In conclusion, our finding that increasing the IL-1Ra expression by infiltrating leukocytes promotes neuroprotection, and that post-stroke injection of IL-1Ra-producing BM cells amplifies endogenous IL-1Ra production, suggests that identifying a way to increase the IL-1Ra expression in leukocytes may be beneficial in future stroke therapy.

## Electronic supplementary material

Below is the link to the electronic supplementary material.

Fig. S1. Control reactions for ISH and IHC. (a-d) *ISH* on parallel sections showing GAPDH mRNA (a), IL-1RA mRNA (b), IL-1α mRNA (c), and IL-1β mRNA (d) (left column), with probe specificity tested using a 100-fold excess of unlabeled probe (a, c, d, middle column), RNAse A pre-treatment (a, c, d, right column), and use of tissue from IL-1Ra-KO mice (b, middle column). IHC controls exemplified in (b, right column) show parallel sections incubated with either goat serum or goat-anti-IL-1Ra antibody. Scale bars: 300 µm (left column), and 300 µm (middle and right column) (TIFF 28392 kb)

Fig. S2. Temporal profile of IL-1R1 and IL-1R2 mRNA and protein. (a and c) Quantitative PCR showing the temporal changes in IL-1RI (a) and IL-1RII (c) mRNA in C57BL/6 mice after pMCAo, including groups of non-lesioned controls (Ctl) and sham mice, n=10-12/group. Cytokine mRNA levels were normalized to a pool of non-lesioned brains. Statistical data are presented as means ± SD (Kruskal-Wallis test with Dunns post-hoc test). *P<0.05, **P<0.01, ***P<0.001. (b and d) IHC staining showing expression of IL-1R1 on microvessels and microglial-like cells 6 and 24 h after pMCAo (c) and leukocyte expression of IL-1RII (d). Scale bars: 20 µm (b, d), and 50 µm (b bottom) (TIFF 1828 kb)

Fig. S3. Sensitivity of cytokine detection using flow cytometry. (a-d) Histograms and dot plots, including control isotype quadrants, of IL-1Ra^+^ (a), IL-1 α ^+^ (b) and IL-1β^+^ (c) CD11b^+^CD45^dim^ microglia or CD11b^+^CD45^high^ leukocytes isolated from the cortex of C57BL/6 non-lesioned control (Ctl) mice and C57BL/6 mice 6, 12 and 24 h after pMCAo, n=4-8/group. Fluorescence double staining of GFP^+^ BM-chimeric mice, shows that IL-1Ra is expressed by microglia, and only rarely by infiltrating leukocytes, 24 h after pMCAo (a). GFP BM-chimeric mice showed IL-1α expression by microglia, but not infiltrating leukocytes, 24 h after pMCAo (b). GFP BM-chimeric mice showed IL-1β expression by both microglia and infiltrating leukocytes 24 h after pMCAo (c). Dot plots, including control isotype quadrants of IL-1Ra^+^/IL-1 α ^+^ and IL-1β ^+^/IL-1α^+^ co-expressing microglia (d), Scale bars: 20 (a-c), and 100 µm (c, right) (TIFF 84404 kb)

Fig S4. Mitogen-activated protein kinase (MAPK) signaling pathways. (a-c) Western blotting showing phospho- (p-) p38 (a) , p-JNK (b) and p-ERK (c) in non-lesioned controls (Ctl) in addition to LM, LM–LM and Tg–LM mice 6 hours after pMCAo (n = 2/group). TFIIB: Transcription factor II B (TIFF 12662 kb)

Fig. S5. Physiological parameters in mice with 5 days survival after pMCAo. (a) Body temperature of LM, LM–LM and Tg–LM mice prior to (0 h) and 30 min, 1 and 3 h after pMCAo. (b to i) Blood gasses (b, c), pH (d), electrolytes (e-g), glucose (h) and lactate (i) measured in pMCAo-operated LM, LM–LM and Tg–LM mice and non-lesioned controls n = 6-8/group. (j) Weight of LM, LM–LM and Tg–LM mice prior to (baseline) and 5 days after pMCAo n = 10-17/group. Statistical data are presented as means ± SD (Kruskal-Wallis test with Dunns post-hoc test) (a-i) or Wilcoxon matched pairs test (i). **P<0.01. Glu, glucose; Lac, lactate (TIFF 60988 kb)

Fig. S6. IL-1Ra mRNA 5 days after pMCAo. (a) IL-1Ra mRNA hybridized cells (arrows) located in peri-infarct and infarct 5 days after pMCAo in Tg–LM mice. (b) Quantitative PCR showing IL-1Ra mRNA in LM, LM–LM and Tg–LM mice 5 days after pMCAo, n = 10/group. Statistical data are presented as means ± SD. Kruskal-Wallis test with Dunns post-hoc test showed no statistical significance. Scale bars: 100 µm (a), and 10 µm (insert) (TIFF 9755 kb)

Fig. S7. IL-1RA, Iba1, CD45, CD68 and GFAP in infarcted human cortex. IHC staining for IL-1RA, Iba1, CD45, CD68 and GFAP was performed on parallel sections from infarcted cortex, 24h post-stroke. Scale bar: 50 µm7 (TIFF 9303 kb)

Table S1. Mouse groups used for assessment of the effect of IL-1Ra and IL-1Ra producing cells on infarct volume (DOCX 20 kb)

Table S2. Human post-stroke scorings (DOCX 18 kb)

Table S3. Antibodies applied for flow cytometry and immunohistochemistry 10 (DOC 40 kb)

Table S4. Correlation analysis of cytokines and cytokine receptor transcript levels 11 (DOC 36 kb)

Table S5. Quantification of IL-1Ra^+^, IL-1α^+^ and IL-1β^+^ cells 12 (DOCX 17 kb)

Table S6. Hargreaves test on tMCAo mice 13 (DOCX 18 kb)

Table S7. Open field test on tMCAo mice 14 (DOCX 23 kb)
