# Supplementary figures and images for: Cell therapy centered on IL-1Ra is neuroprotective in experimental stroke
Source: Acta Neuropathol. 2016 Feb 9;131:775–91. doi: 10.1007/s00401-016-1541-5 (PMC4835531; doi:10.1007/s00401-016-1541-5)

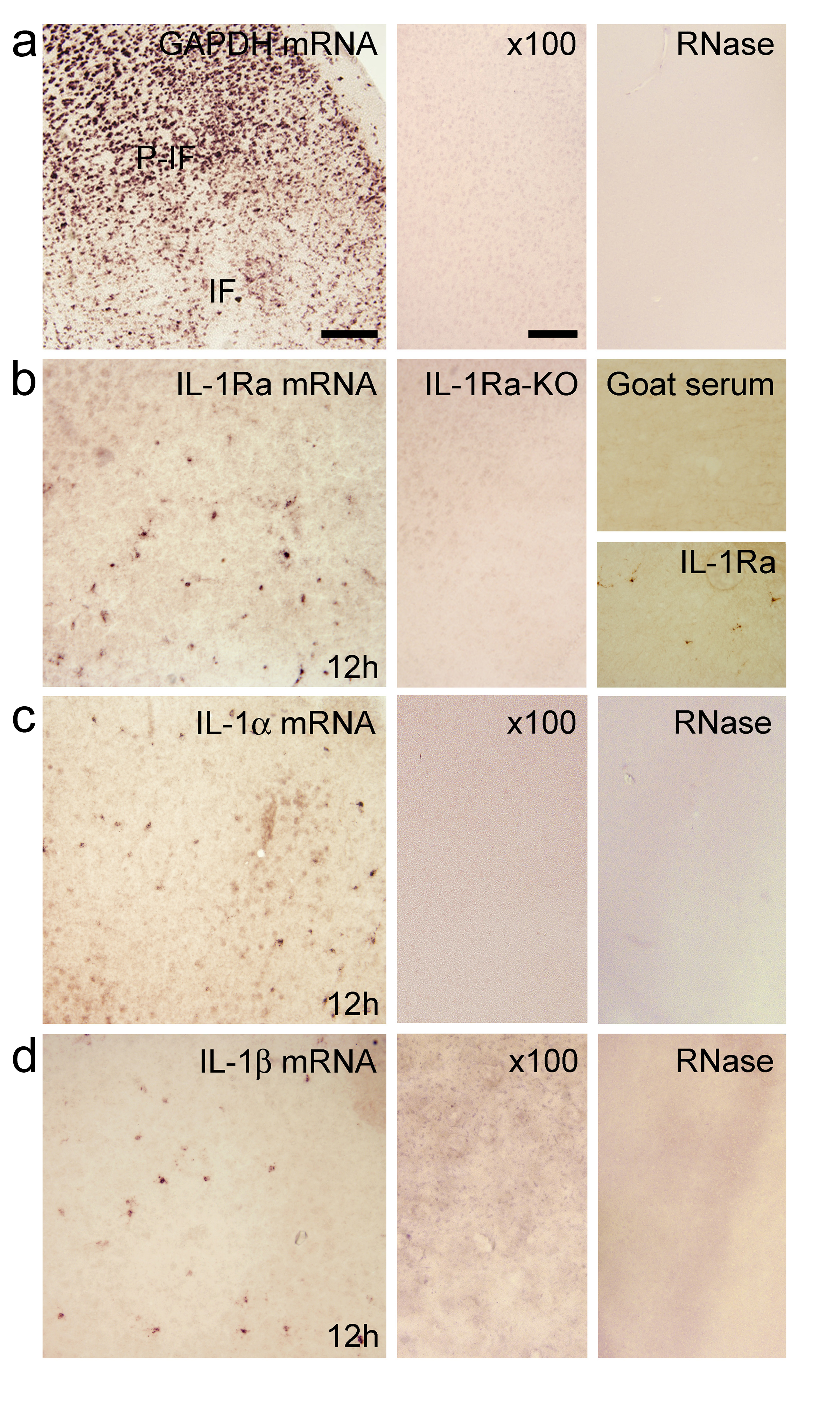

Supplement: Supplementary file 1 — Fig. S1. Control reactions for ISH and IHC. (a-d) ISH on parallel sections showing GAPDH mRNA (a), IL-1RA mRNA (b), IL-1α mRNA (c), and IL-1β mRNA (d) (left column), with probe specificity tested using a 100-fold excess of unlabeled probe (a, c, d, middle column), RNAse A pre-treatment (a, c, d, right column), and use of tissue from IL-1Ra-KO mice (b, middle column). IHC controls exemplified in (b, right column) show parallel sections incubated with either goat serum or goat-anti-IL-1Ra antibody. Scale bars: 300 µm (left column), and 300 µm (middle and right column) (TIFF 28392 kb) [file 401_2016_1541_MOESM1_ESM.tif]

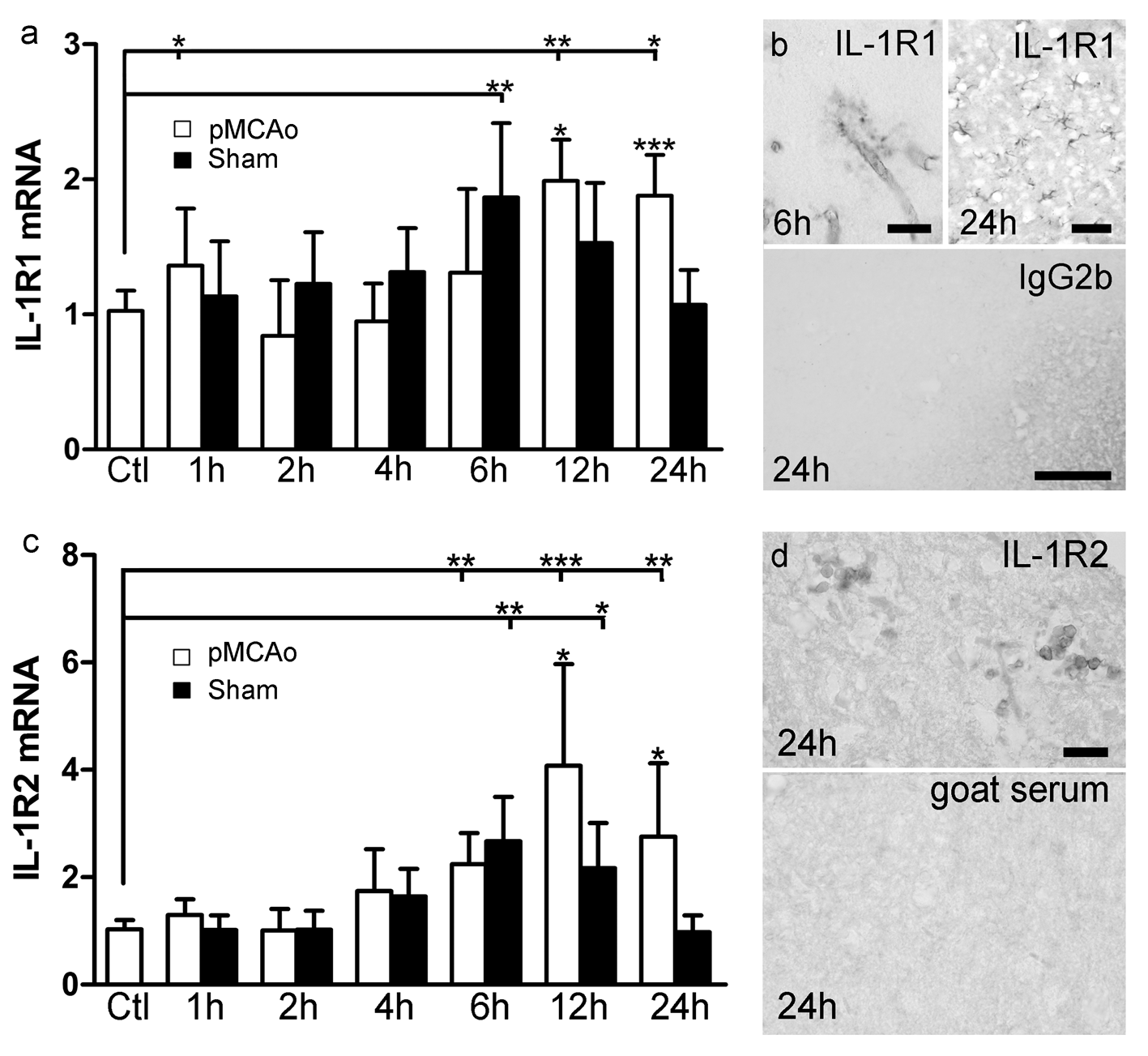

Supplement: Supplementary file 2 — Fig. S2. Temporal profile of IL-1R1 and IL-1R2 mRNA and protein. (a and c) Quantitative PCR showing the temporal changes in IL-1RI (a) and IL-1RII (c) mRNA in C57BL/6 mice after pMCAo, including groups of non-lesioned controls (Ctl) and sham mice, n=10-12/group. Cytokine mRNA levels were normalized to a pool of non-lesioned brains. Statistical data are presented as means ± SD (Kruskal-Wallis test with Dunns post-hoc test). *P<0.05, **P<0.01, ***P<0.001. (b and d) IHC staining showing expression of IL-1R1 on microvessels and microglial-like cells 6 and 24 h after pMCAo (c) and leukocyte expression of IL-1RII (d). Scale bars: 20 µm (b, d), and 50 µm (b bottom) (TIFF 1828 kb) [file 401_2016_1541_MOESM2_ESM.tif]

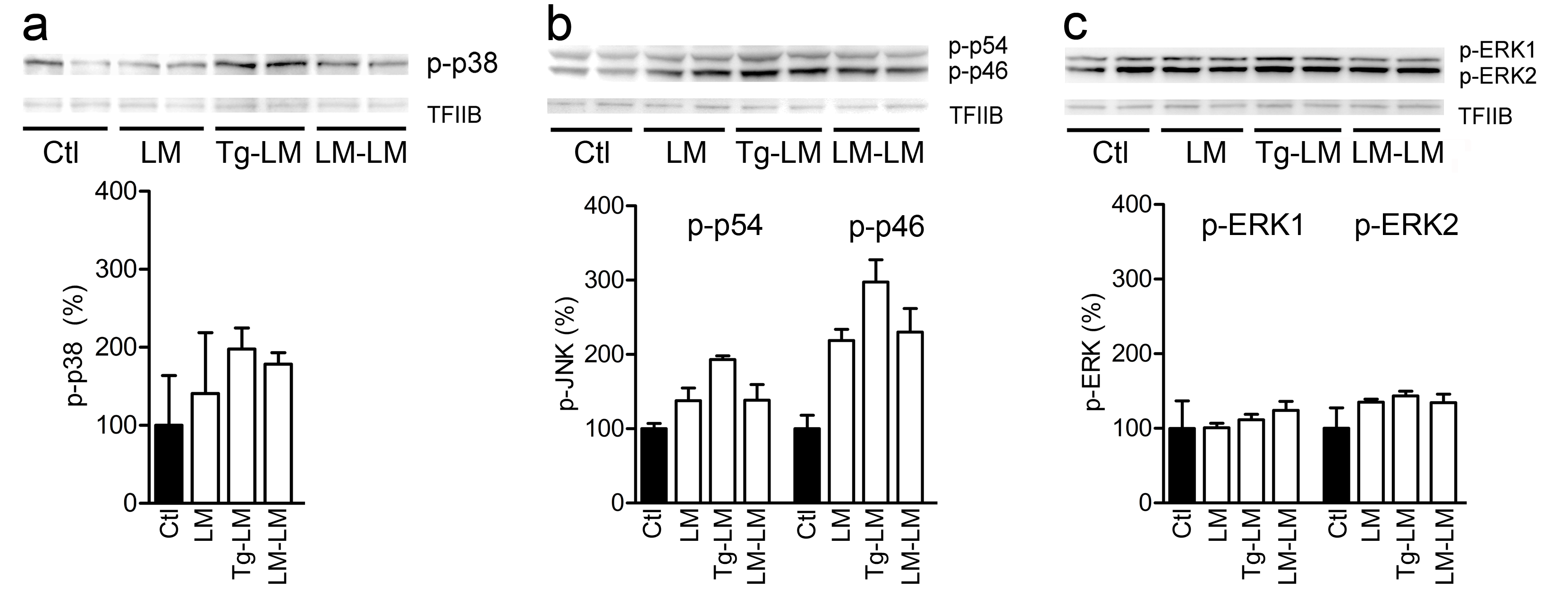

Supplement: Supplementary file 4 — Fig S4. Mitogen-activated protein kinase (MAPK) signaling pathways. (a-c) Western blotting showing phospho- (p-) p38 (a) , p-JNK (b) and p-ERK (c) in non-lesioned controls (Ctl) in addition to LM, LM–LM and Tg–LM mice 6 hours after pMCAo (n = 2/group). TFIIB: Transcription factor II B (TIFF 12662 kb) [file 401_2016_1541_MOESM4_ESM.tif]

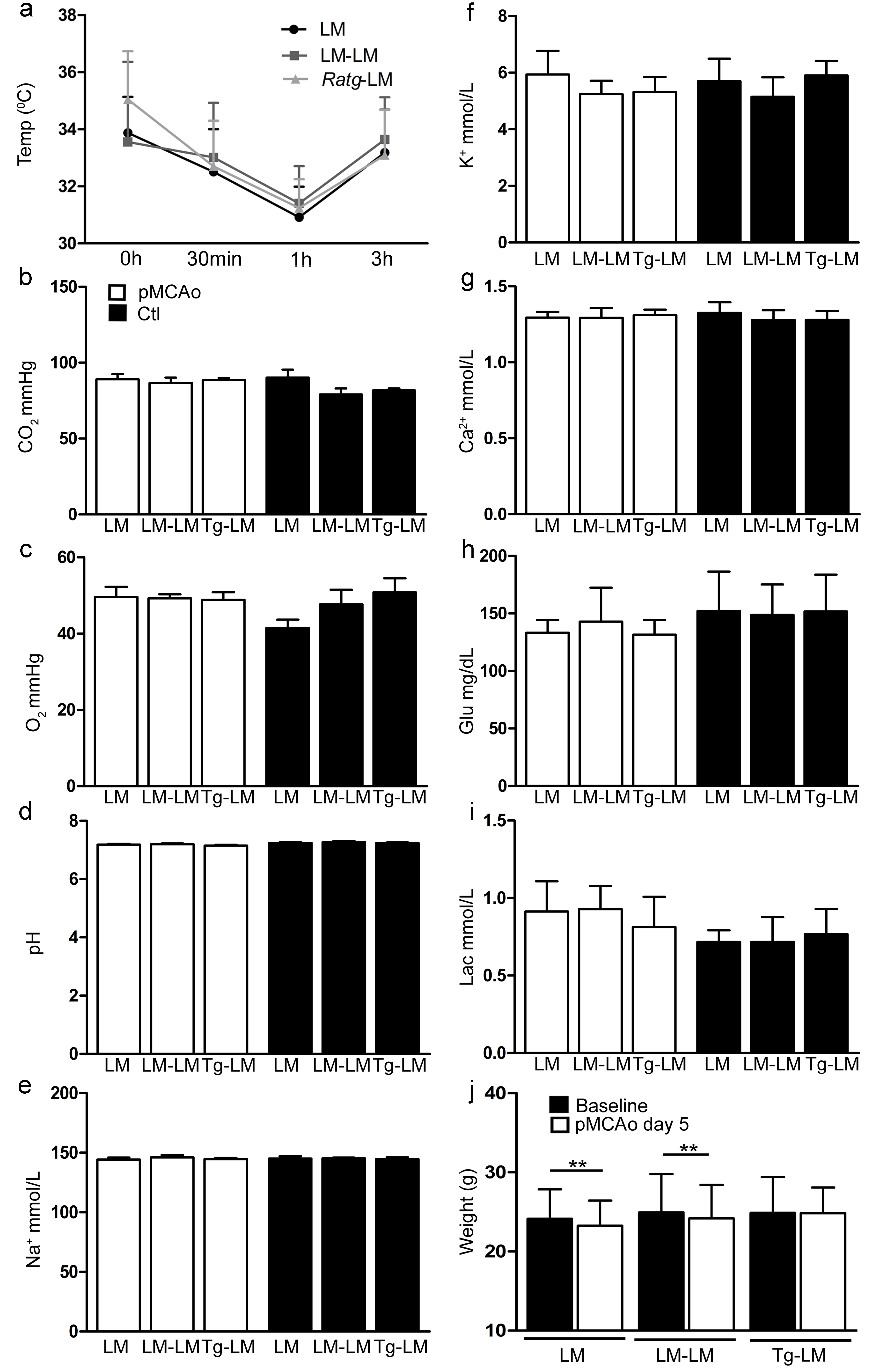

Supplement: Supplementary file 5 — Fig. S5. Physiological parameters in mice with 5 days survival after pMCAo. (a) Body temperature of LM, LM–LM and Tg–LM mice prior to (0 h) and 30 min, 1 and 3 h after pMCAo. (b to i) Blood gasses (b, c), pH (d), electrolytes (e-g), glucose (h) and lactate (i) measured in pMCAo-operated LM, LM–LM and Tg–LM mice and non-lesioned controls n = 6-8/group. (j) Weight of LM, LM–LM and Tg–LM mice prior to (baseline) and 5 days after pMCAo n = 10-17/group. Statistical data are presented as means ± SD (Kruskal-Wallis test with Dunns post-hoc test) (a-i) or Wilcoxon matched pairs test (i). **P<0.01. Glu, glucose; Lac, lactate (TIFF 60988 kb) [file 401_2016_1541_MOESM5_ESM.tif]

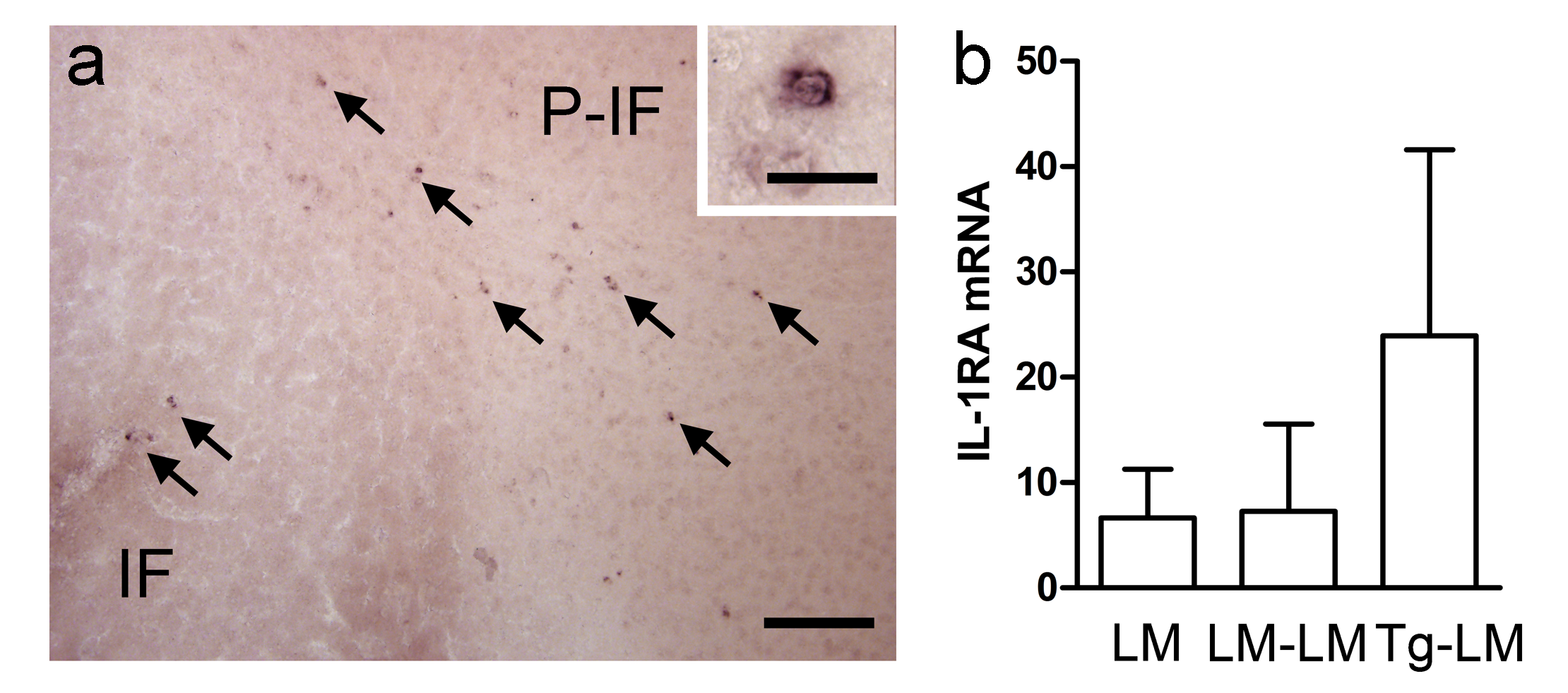

Supplement: Supplementary file 6 — Fig. S6. IL-1Ra mRNA 5 days after pMCAo. (a) IL-1Ra mRNA hybridized cells (arrows) located in peri-infarct and infarct 5 days after pMCAo in Tg–LM mice. (b) Quantitative PCR showing IL-1Ra mRNA in LM, LM–LM and Tg–LM mice 5 days after pMCAo, n = 10/group. Statistical data are presented as means ± SD. Kruskal-Wallis test with Dunns post-hoc test showed no statistical significance. Scale bars: 100 µm (a), and 10 µm (insert) (TIFF 9755 kb) [file 401_2016_1541_MOESM6_ESM.tif]

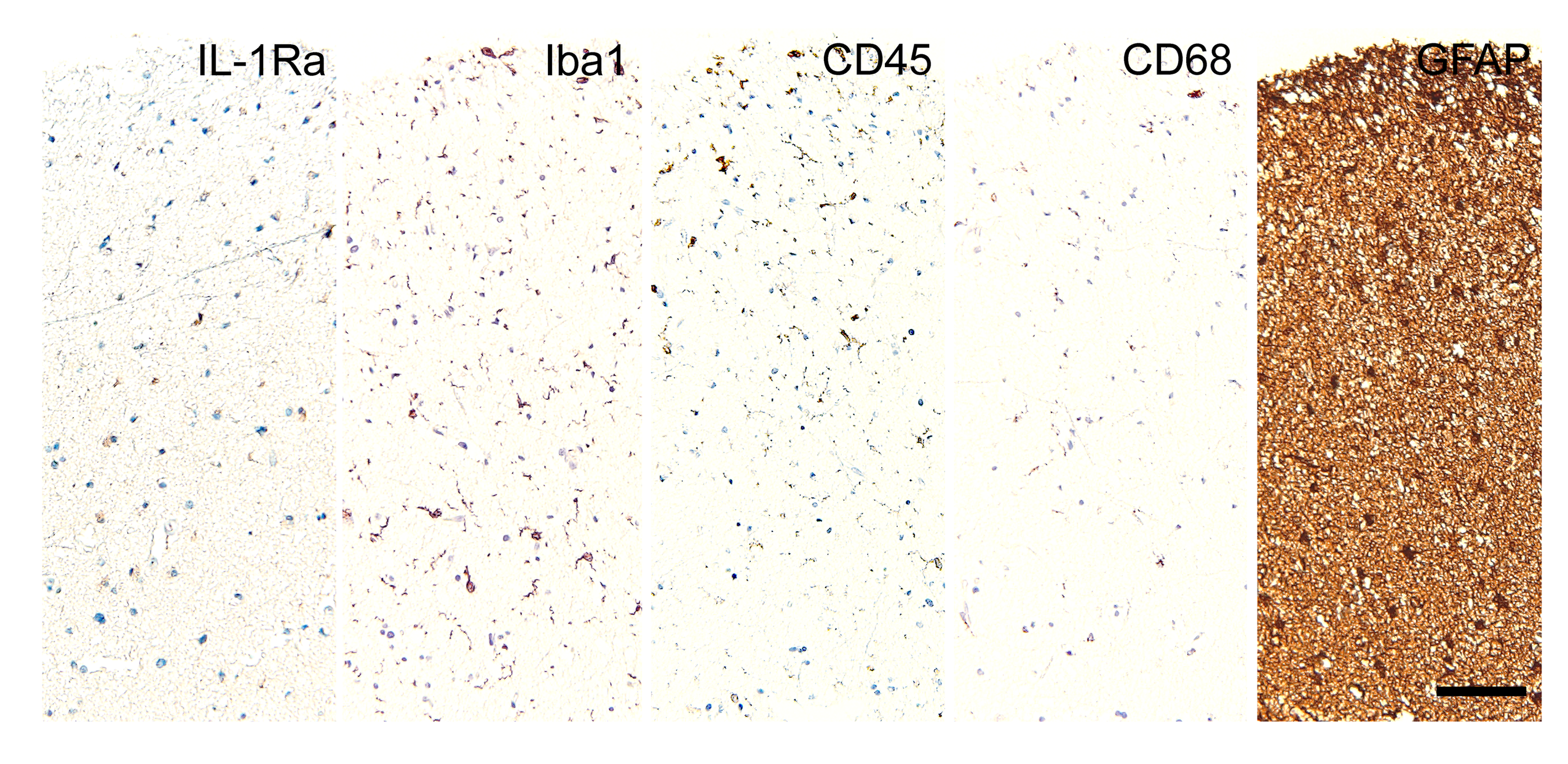

Supplement: Supplementary file 7 — Fig. S7. IL-1RA, Iba1, CD45, CD68 and GFAP in infarcted human cortex. IHC staining for IL-1RA, Iba1, CD45, CD68 and GFAP was performed on parallel sections from infarcted cortex, 24h post-stroke. Scale bar: 50 µm7 (TIFF 9303 kb) [file 401_2016_1541_MOESM7_ESM.tiff]
