# Supplementary material for: Cell therapy centered on IL-1Ra is neuroprotective in experimental stroke
Source: Acta Neuropathol. 2016 Feb 9;131:775–91. doi: 10.1007/s00401-016-1541-5 (PMC4835531; doi:10.1007/s00401-016-1541-5)
Supplement: Supplementary file 8 — Table S1. Mouse groups used for assessment of the effect of IL-1Ra and IL-1Ra producing cells on infarct volume (DOCX 20 kb) [file 401_2016_1541_MOESM8_ESM.docx]

**Table S1.** Mouse groups used for assessment of effect of IL-1Ra and IL-1Ra overproducing BM cells in experimental stroke

| **Experiment** | **Details on mice**  **&**  **BM cells** | **Stroke model** | **Survival time** | **Mouse**  **Number of mice**  **(n)** | | | **Total number of mice** |
| --- | --- | --- | --- | --- | --- | --- | --- |
| Effect of IL-1Ra deficiency or overexpression | IL-1Ra deficiency | pMCAo | 24 h | IL-1Ra KO  (n=14) | LM  (n=14) | - | 28 |
|  | IL-1Ra overexpression | pMCAo | 24 h | IL-1Ra Tg  (n=14) | LM  (n=14) | - | 28 |
| Effect of BM chimerism using whole body irradiated B6 recipients | Donor BM cells from B6, IL-1Ra-KO and IL-1Ra-Tg mice | pMCAo | 24 h | B6 - B6^*^  (n= 12) | KO - B6^*^  (n=14) | Tg - B6^*^  (n=12) | 38 |
| Therapeutic effect of post-stroke treatment (30 min after arterial occlusion) | BM cells from IL-1Ra Tg and LM mice | pMCAo | 24 h | IL-1Ra - LM  (n=14) | LM - LM  (n=14) | - | 28 |
|  |  | pMCAo | 5 d | LM  (n=14) | LM - LM  (n=14) | Tg - LM  (n=14) | 42 |
| Therapeutic effect of post-stroke treatment (30 min after filament removal) | BM cells from IL-1Ra Tg and LM mice | tMCAo | 24 h | LM  (n=12 ) | LM - LM  (n=15) | Tg - LM  (n= 17) | 34 |

LM, littermate wildtype; B6, C57BL/6. Asterisks indicate that the B6 recipient mouse is whole body irradiated.
