# Supplementary material for: Cell therapy centered on IL-1Ra is neuroprotective in experimental stroke
Source: Acta Neuropathol. 2016 Feb 9;131:775–91. doi: 10.1007/s00401-016-1541-5 (PMC4835531; doi:10.1007/s00401-016-1541-5)
Supplement: Supplementary file 9 — Table S2. Human post-stroke scorings (DOCX 18 kb) [file 401_2016_1541_MOESM9_ESM.docx]

**Table S2.** Human post-stroke scorings

| **Case reports**  **Nr.** | **Sex** | **Age** | **Infarcted brain area** | **Infarct age** | **Score** | | |
| --- | --- | --- | --- | --- | --- | --- | --- |
|  |  |  |  |  | **IF** | **p-IF** | **Normal** |
| **#1** | M | 61 | Right parietal lobe | 1 day | +++ | +++ | - |
| **#2** | M | 68 | Left parietal lobe | 1 day | ++ | +++ | - |
| **#3** | F | 80 | Right frontal lobe | 1 day | - | +++++ | ++ |
| **#4** | F | 80 | Pons | 1 day | ++ | ++++ | + |
| **#5** | F | 67 | Right occipital lobe | 1 day | + | + | - |
| **#6** | F | 78 | Right frontoparietal lobe | ≤ 2 days | - | ++++ | - |
| **#7** | F | 83 | Right hippocampus | ≤ 2 days | ´- | + | - |
| **#8** | F | 67 | Right occipital lobe | ≤ 2 days | + | + | - |
| **#9** | F | 83 | cerebellum | < 5 days | - | ++ | + |
| **#10** | F | 80 | Medulla oblongata | < 5 days | - | ++ | - |
| **#11** | M | 38 | Right temporal lobe | < 5 days | ++ | +++ | - |
| **#12** | M | 76 | Left temporal lobe | ≥ 7 days | +++ | ++++ | + |
| **#13** | F | 73 | Right temporal lobe | ≥ 7 days | + | ++ | + |
| **#14** | M | 67 | Left temporal lobe | ≥ 7 days | +++ | + | - |
| **#15** | M | 61 | Right parietal lobe | ≥ 7 days | +++ | +++ | - |
| **#16** | F | 80 | Striatum | ≥ 7 days | ++++ | +++++ | + |
| **#17** | M | 68 | Caudate nucleus | ≥ 7 days | +++ | ++ | + |
| **#18** | M | 68 | Insula | ≥ 7 days | ++++ | +++++ | + |
| **#19** | M | 59 | Right parietal lobe | ≥ 7 days | ++++ | ++++ | - |
| **#20** | M | 57 | Left internal capsule | ≥ 7 days | +++ | +++++ | + |
| **#21** | M | 48 | Right temporal lobe | ≥ 7 days | + | ++ | + |
