# Supplementary material for: Cell therapy centered on IL-1Ra is neuroprotective in experimental stroke
Source: Acta Neuropathol. 2016 Feb 9;131:775–91. doi: 10.1007/s00401-016-1541-5 (PMC4835531; doi:10.1007/s00401-016-1541-5)
Supplement: Supplementary file 10 — Table S3. Antibodies applied for flow cytometry and immunohistochemistry 10 (DOC 40 kb) [file 401_2016_1541_MOESM10_ESM.doc]

| **Method** |  | **Antibodies &Reagents** | **Clone** | **Source** |
| --- | --- | --- | --- | --- |
| **Flow cytometry**  **Immunohistochemistry** | **Primary**  **antibodies**  **Controls**  **Secondary**  **antibodies**  **Primary**  **antibodies**  **Controls**  **Secondary**  **antibodies** | CD11b-PerCP  CD45-PE  GFAP-488  IL-1Ra  IL-1α-Biotin  IL-1β  Rat IgG2b-PE  Rat IgG2b-PerCP  Mouse IgG1-488  Rat IgG2b-FITC  IgG fraction  IgG fraction-Biotin  Rat IgG2a  Alexa-647 α-rabbit  Alexa-647 α-rat  Alexa-488 α-rat  Strep-FITC  IL-1Ra  IL-1Ra  IL-1β  IL-1α Biotin  IL-1α  IL-1RI  IL-1RII  CD11b  CD45 (human)  Iba1 (human)  CD68 (human)  GFAP  CD41  IgG fraction  IgG2b  IgG2a  Serum  Alexa-546 α-rat  Alexa-488 α-goat  Alexa-594 α-rabbit  Alexa-546 α-rabbit  Alexa-488 α-rat  Streptavidin-HRP | M1/70 (Rat IgG2b)  30-F11(Rat IgG2b)  131-17719 ( Mouse IgG1)  40007 (Rat IgG2a)  Polyclonal Rabbit  Polyclonal Rabbit  A95-1 (Rat IgG2b)  A95-1 (Rat IgG2b)  MOPC-21 (Mouse IgG1)  A95-1 (Rat IgG2b)  Polyclonal Rabbit  Polyclonal Rabbit  DD13 (Rat IgG2a)  Polyclonal Goat  Polyclonal Goat  -  -  Polyclonal Rabbit  40007 (Rat IgG2a)  Polyclonal Rabbit  Polyclonal Rabbit  Polyclonal Goat  RMML1-2 (Rat IgG2a)  Polyclonal Goat  5C6 (IgG2b)  2B11 (IgG1)  Polyclonal Rabbit  PG-M1 (IgG3)  Polyclonal Rabbit  MWReg30 (Rat IgG1)  Polyclonal Rabbit  RTK4530  RTK2758  Goat  Goat  Chicken  Donkey  Goat  Donkey  - | BD Biosciences, #550993  BD Biosciences, #553081  Invitrogen, # A21294  R&D Systems, MAB480  Serotec, # AAM31B  Serotec, #AAM13G  BD Biosciences, # 553989  BD Biosciences, #550764  Caltag, # MG120  BD Biosciences, #556923  DakoCytomation, # X0903  Santa Cruz Biotechnology, # Sc-2763  Biocompare, # P54605M  Invitrogen, #A21244  Invitrogen, # A21247  Invitrogen, #A21208  BD Biosciences, #554060  R&D Systems, #AF-480-NA  R&D Systems, #MAB480  Serotec, #AAM13G  Serotec, #AAM31B  R&D Systems, #AB-400-NA  Serotec, #MCA1761Z  R&D systems, #AF563  Serotec, #MCA711  Dako, #M0701  Wako, #019-19741  Dako, # M0876  Dako, #Z0334  Serotec, # MCA2245GA  DakoCytomation, # X0903  BioLegend, #400601  BioLegend, #400501  DakoCytomation, # X0907  Invitrogen, #A21085  Invitrogen, #A21467  Invitrogen, #A21207  Invitrogen, #A11010  Invitrogen, #A21208  DakoCytomation, # P0397 |

**Table S3.** Antibodies applied for flow cytometry and immunohistochemistry
