# Supplementary material for: Cell therapy centered on IL-1Ra is neuroprotective in experimental stroke
Source: Acta Neuropathol. 2016 Feb 9;131:775–91. doi: 10.1007/s00401-016-1541-5 (PMC4835531; doi:10.1007/s00401-016-1541-5)
Supplement: Supplementary file 11 — Table S4. Correlation analysis of cytokines and cytokine receptor transcript levels 11 (DOC 36 kb) [file 401_2016_1541_MOESM11_ESM.doc]

**Table S4.** Correlation analysis of cytokines and cytokine receptor transcript levels

| Spearman test (r_s_) | Control  (n=11) | 1 hour  (n=10) | 2 hours  (n=10) | 4 hours  (n=10) | 6 hours  (n=10) | 12 hours  (n=9) | 24 hours  (n=10) |
| --- | --- | --- | --- | --- | --- | --- | --- |
| IL-1ra mRNA vs. IL-1β mRNA | P>0.6  (-0.2) | P<0.009  (0.8) | P<0.006  (0.8) | P>0.9  (0.03) | P<0.03  (0.7) | P<0.002  (0.9) | P<0.02  (0.8) |
| IL-1ra mRNA vs.  IL-1α mRNA | P>0.2  (0.3) | P>0.3  (0.3) | P>0.6  (0.2) | P>0.9  (0.03) | P>0.5  (-0.2) | P>0.7  (0.1) | P<0.003  (0.9) |
| IL-1β mRNA vs.  IL-1α mRNA | P>0.6  (-0.2) | P>0.9  (-0.01) | P>0.5  (0.2) | P>0.3  (-0.4) | P>0.2  (-0.4) | P>0.9  (0.02) | P>0.8  (0.09) |
| IL-1β mRNA vs.  IL-1RI mRNA | P>0.5  (0.2) | P>0.5  (0.2) | P>0.2  (0.4) | P>0.9  (0.01) | P>0.1  (0.5) | P>0.8  (-0.05) | P<0.002  (0.9) |
| IL-1β mRNA vs.  IL-1RII mRNA | P>0.5  (0.2) | P>0.6  (0.2) | P> 0.4  (0.3) | P>0.9  (0.01) | P>0.7  (0.1) | P>0.3  (0.4) | P<0.006  (0.8) |
| IL-1α mRNA vs.  IL-1RI mRNA | P>0.8  (-0.08) | P<0.01  (0.8) | P>0.7  (-0.1) | P>0.2  (0.4) | P>0.3  (0.3) | P<0.04  (0.7) | P>0.08  (0.6) |
| IL-1α mRNA vs.  IL-1RII mRNA | P>0.8  (-0.01) | P>0.3  (0.3) | P>0.9  (0.04) | P>0.8  (-0.06) | P<0.04  (0.7) | P<0.01  (0.8) | P<0.01  (0.8) |
