# Supplementary material for: Cell therapy centered on IL-1Ra is neuroprotective in experimental stroke
Source: Acta Neuropathol. 2016 Feb 9;131:775–91. doi: 10.1007/s00401-016-1541-5 (PMC4835531; doi:10.1007/s00401-016-1541-5)
Supplement: Supplementary file 12 — Table S5. Quantification of IL-1Ra+, IL-1α+ and IL-1β+ cells 12 (DOCX 17 kb) [file 401_2016_1541_MOESM12_ESM.docx]

|  |  | **IL-1α** | |  | **IL-1β** | |  | **IL-1Ra** | |
| --- | --- | --- | --- | --- | --- | --- | --- | --- | --- |
| **Survival** | **n** | **Microglia** | **Leukocytes** | **n** | **Microglia** | **Leukocytes** | **n** | **Microglia** | **Leukocytes** |
| **Ctl** | 4 | 321 ± 269* | 26 ± 23 | 5 | 1300 ± 433** | 89 ± 65 | 5 | 6613 ± 2244** | 65 ± 44 |
| **6h** | 4 | 559 ± 78* | 69 ± 31 | 5 | 1022 ± 494* | 192 ± 117 | 5 | 8817 ± 453* | 597 ± 344 |
| **12h** | 4 | 1954 ± 264* | 135 ± 43 | 7 | 6371 ± 215* | 152 ± 152 | 7 | 5740 ± 2090* | 169 ± 171 |
| **24h** | 4 | 4072 ± 368* | 48 ± 9 | 9 | 11374 ± 2032 | 6327 ± 3297 | 9 | 20408 ± 7213*** | 190 ± 157 |

**Table S5.** Quantification of IL-1Ra^+^, IL-1α^+^ and IL-1β^+^ cells
