# Supplementary material for: Cell therapy centered on IL-1Ra is neuroprotective in experimental stroke
Source: Acta Neuropathol. 2016 Feb 9;131:775–91. doi: 10.1007/s00401-016-1541-5 (PMC4835531; doi:10.1007/s00401-016-1541-5)
Supplement: Supplementary file 13 — Table S6. Hargreaves test on tMCAo mice 13 (DOCX 18 kb) [file 401_2016_1541_MOESM13_ESM.docx]

**Table S6.** Hargreaves test on tMCAo mice

| **Hargreaves** | **No of mice**  (n) | **Unlesioned** | | **tMCAo** | | | | | |
| --- | --- | --- | --- | --- | --- | --- | --- | --- | --- |
|  |  |  |  | **LM** | | **LM-LM** | | **Tg-LM** | |
|  |  | Left vs. Right | | Left vs. Right | | Left vs. Right | | Left vs. Right | |
|  |  | Mean ± SD | P ≤ | Mean ± SD | P ≤ | Mean ± SD | P ≤ | Mean ± SD | P ≤ |
| Pretraining  (s) | 50 | 3.2 ± 1.8 /  3.5 ± 1.5 | ns | *-* | *-* | *-* | *-* | *-* | *-* |
| Asymmetry  (Δs) | 41 | - | - | -1.9 ± 1.6 /  -0.4 ± 1.3 | * | -1.9 ± 1.9 /  -0.8 ± 1.3 | ** | -0.7 ± 2.1 /  -0.4 ± 2.1 | ns |

Ns, non-significant.
