# Supplementary material for: Cell therapy centered on IL-1Ra is neuroprotective in experimental stroke
Source: Acta Neuropathol. 2016 Feb 9;131:775–91. doi: 10.1007/s00401-016-1541-5 (PMC4835531; doi:10.1007/s00401-016-1541-5)
Supplement: Supplementary file 14 — Table S7. Open field test on tMCAo mice 14 (DOCX 23 kb) [file 401_2016_1541_MOESM14_ESM.docx]

**Table S7.** Open field test on tMCAo mice

|  |  |  |  |  |  | **tMCAO** |  |  |
| --- | --- | --- | --- | --- | --- | --- | --- | --- |
| **Open Field Test** | **n** | **Non-lesioned (Ctl)** |  | **LM** |  | **LM-LM** |  | **Tg-LM** |
|  |  | Mean ± SD | P ≤ | Mean ± SD | P ≤ | Mean ± SD | P ≤ | Mean ± SD |
| **Horizontal activity** |  |  |  |  |  |  |  |  |
| Total distance (m) | 9 | 27.5 ± 3.6 | ** | 20.3 ± 6.2 | ## | 30.9 ± 7.6 | # | 29.5 ± 4.2 |
| Mean velocity (cm/sec) | 9 | 45.9 ± 6.0 | ** | 33.9 ± 10.3 | ## | 51.7 ± 12.6 | # | 49.3 ± 7.1 |
| Number of zone changes | 9 | 147.3 ± 44.5 | ns | 114.6 ± 68.8 | ns | 150.8 ± 65.9 | ns | 131.2 ± 56.4 |
| **Vertical activity** |  |  |  |  |  |  |  |  |
| Wall rearings | 9 | 39.4 ± 16.6 | * | 22.7 ± 14.8 | # | 51.9 ± 20.8 | # | 54.9 ± 24.3 |
| Center rearings | 9 | 47.0 ± 19.9 | 0.09 | 23.9 ± 21.4 | ns | 24.1 ± 15.4 | ☼ | 55.9 ± 27.5 |
| Jumping | 9 | 0.7 ± 1.5 | ns | 0.0 ± 0.0 | # | 4.2 ± 7.8 | ns | 2.3± 6.3 |
| **Anxiety** |  |  |  |  |  |  |  |  |
| Grooming | 9 | 4.6 ± 1.0 | ns | 11.0 ± 8.9 | ns | 8.7 ± 4.7 | ns | 5.0 ± 2.1 |
| Digging | 9 | 0.0 ± 0.0 | * | 8.1 ± 8.8 | ns | 15.8 ± 20.4 | ns | 8.1 ± 5.7 |
| Droppings | 9 | 0.8 ± 1.8 | ns | 2.1 ± 2.5 | ns | 2.2 ± 2.7 | ns | 0.8 ± 1.0 |
| Urinations | 9 | 0.1 ± 0.3 | * | 0.7 ± 0.7 | ns | 0.2 ± 0.4 | ns | 0.2 ± 0.4 |
| Latency to rear (sec.) | 9 | 24.0 ± 13.2 | * | 61.9 ± 58.6 | ns | 36.0 ± 49.0 | ns | 24.2 ± 9.6 |
| Center (s)/total distance (cm) | 9 | 0.003 ± 0.001 | ** | 0.0009 ± 0.0006 | ns | 0.0008 ± 0.0006 | # ☼ | 0.002 ± 0.001 |

Ctl vs. LM (*) (t-test). LM vs. LM-LM or Tg-LM (#) and LM-LM vs. Tg-LM (☼) (ANOVA-test). ns, non-significant.
